# Supplementary material for: Expression of ENL YEATS domain tumor mutations in nephrogenic or stromal lineage impairs kidney development
Source: Nat Commun. 2025 Mar 14;16:2531. doi: 10.1038/s41467-025-57926-z (PMC11909213; doi:10.1038/s41467-025-57926-z)
Supplement: Supplementary file 1 — Supplementary Information [file 41467_2025_57926_MOESM1_ESM.pdf]

Supplementary Figures

Expression of ENL YEATS domain tumor mutations in nephrogenic or stromal lineage impairs kidney development

Zhaoyu Xue[1,#], Hongwen Xuan[1,#], Kin Lau[2], Yangzhou Su[1], Marc Wegener[3], Kuai Li[1], Lisa Turner[4], Marie Adams[3], Xiaobing Shi[1], and Hong Wen[1,*]

Affiliations

[1]Department of Epigenetics, Van Andel Institute, Grand Rapids, MI 49503, USA

[2]Bioinformatics and Biostatistics Core, Van Andel Institute, Grand Rapids, MI 49503, USA

[3]Genomics Core, Van Andel Institute, Grand Rapids, MI 49503, USA

[4]Pathology Core, Van Andel Institute, Grand Rapids, MI 49503, USA

[*]Corresponding author. Email: hong.wen@vai.org

[#]Threse authors contributed equally: Zhaoyu Xue and Hongwen Xuan.

Supplementary Fig. 1

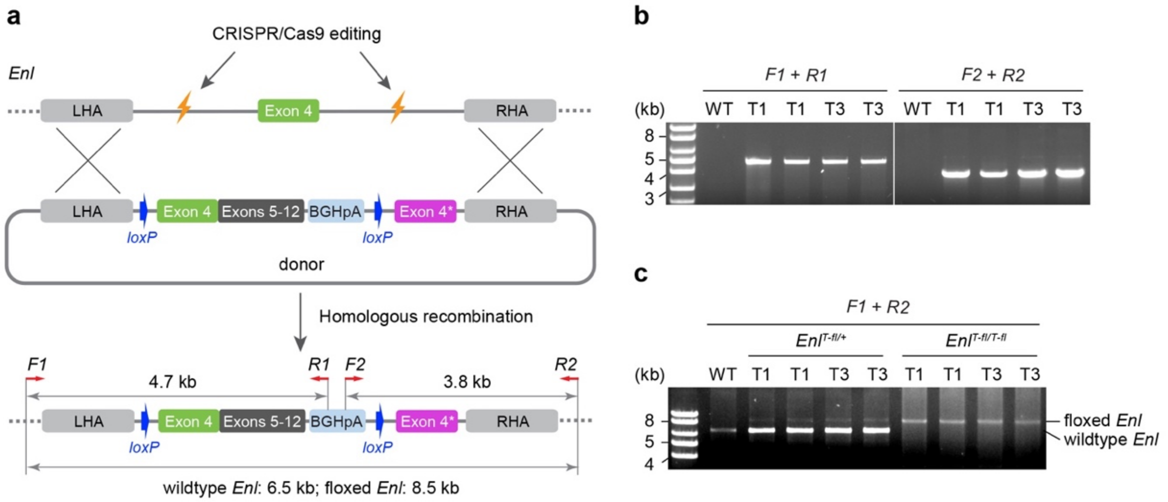

Supplementary Fig. 1 | Generation of conditional knock-in (cKI) *Enl*$^{T\text{-}fl}$ mouse models. a, Schematic of targeting strategy to generate cKI *Enl*$^{T\text{-}fl}$ mouse model and PCR validation strategy. **b**, Long-range PCR validation of the knock-in cassette. **c**, Long-range PCR detection of the wildtype and floxed *Enl* alleles.

Supplementary Fig. 2

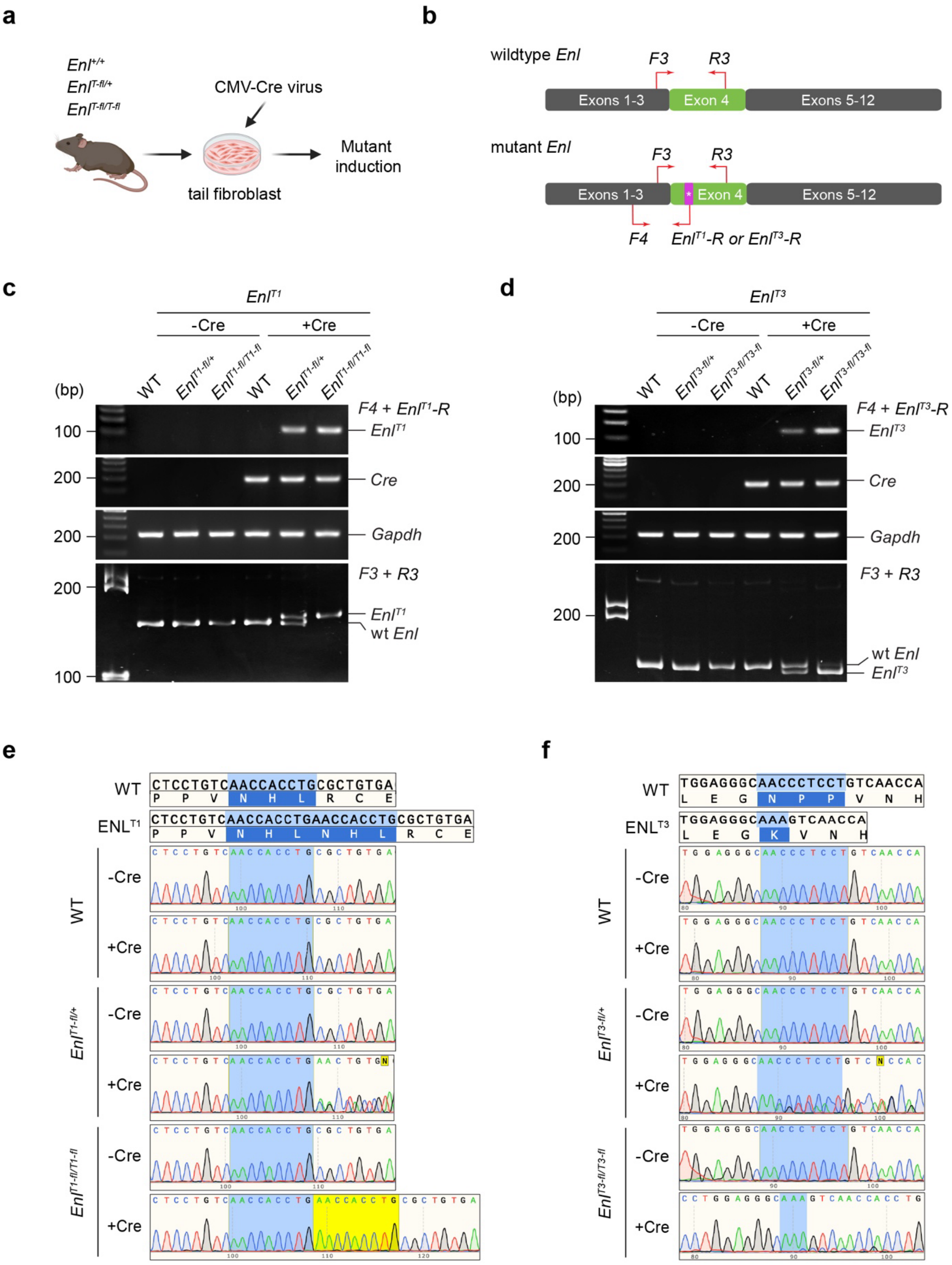

Supplementary Fig. 2 | Mouse model validation. a, Schematic of the experimental design. Tail fibroblasts isolated and cultured from wildtype, *Enl*$^{T\text{-}fl/+}$ and *Enl*$^{T\text{-}fl/T\text{-}fl}$ mice were infected by lentivirus expressing CMV-Cre. Extracted RNA from these cells were used for RT-qPCR validation of the expression of wildtype *Enl* and *Enl*^{T} mutants. Created in BioRender. Wen, H. (2025) https://BioRender.com/c12k604. **b**, Schematic of wildtype and mutant specific PCR strategy. **c**, **d**, RT-PCR validation of the expression of wildtype *Enl*, *Enl*^{T}, *Cre*, and internal control *Gapdh*. Note that in *Enl*$^{T\text{-}fl}$ heterozygous fibroblasts induced by Cre, the expression of wildtype *Enl* and *Enl*^{T1} (**c**) or *Enl*^{T3} (**d**) is at approximately 1:1 ratio. **e**, **f**, Confirmation of *Enl*^{T1} (**e**) and *Enl*^{T3} (**f**) mutant expression upon Cre induction by DNA Sanger sequencing.

Supplementary Fig. 3

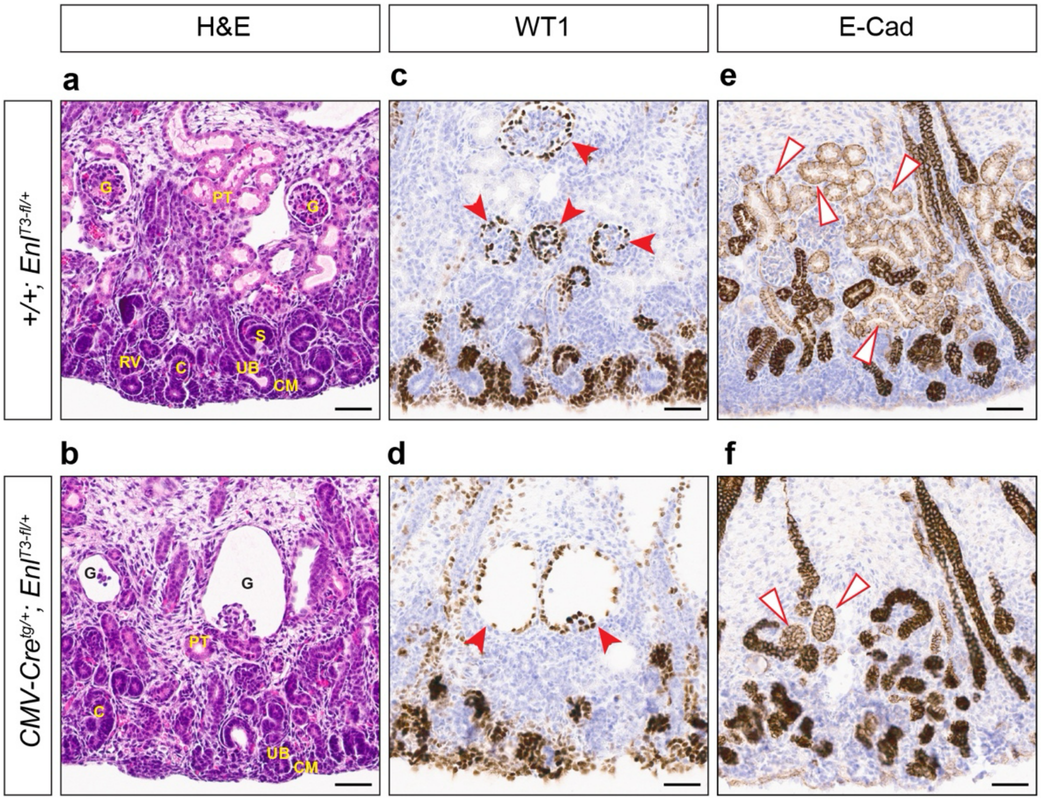

Supplementary Fig. 3 | H&E and immunostaining of kidneys from CMV-Cre driven ENL^{T3} mouse model. a, **b**, H&E staining of kidney sections from E18.5 *Enl*$^{T3\text{-}fl/+}$ (**a**) and *CMV-Cre*$^{tg/+;}$ *Enl*$^{T3\text{-}fl/+}$ (**b**) embryos. Scale bars, 0.5 mm. C, comma-shaped body; CM, cap mesenchyme; G, glomerulus; S, S-shaped body; RV, renal vesicle; PT, proximal tubule; UB, ureteric bud. **c**, **d**, Immunostaining for WT1 of kidney sections from E18.5 *Enl*$^{T3\text{-}fl/+}$ (**c**) and *CMV-Cre*$^{tg/+}$*; Enl*$^{T3\text{-}fl/+}$ (**d**) embryos. Solid red arrowheads indicate developing glomeruli at different stages. **e**, **f**, Immunostaining for E-Cadherin (E-Cad) of kidney sections from E18.5 *Enl*$^{T3\text{-}fl/+}$ (**e**) and *CMV-Cre*$^{tg/+}$*; Enl*$^{T3\text{-}fl/+}$ (**f**) embryos. Red open triangles indicate proximal tubules with light E-Cad staining. Scale bars in **c**-**f**, 50 µm. Similar results were obtained in three independent experiments.

Supplementary Fig. 4

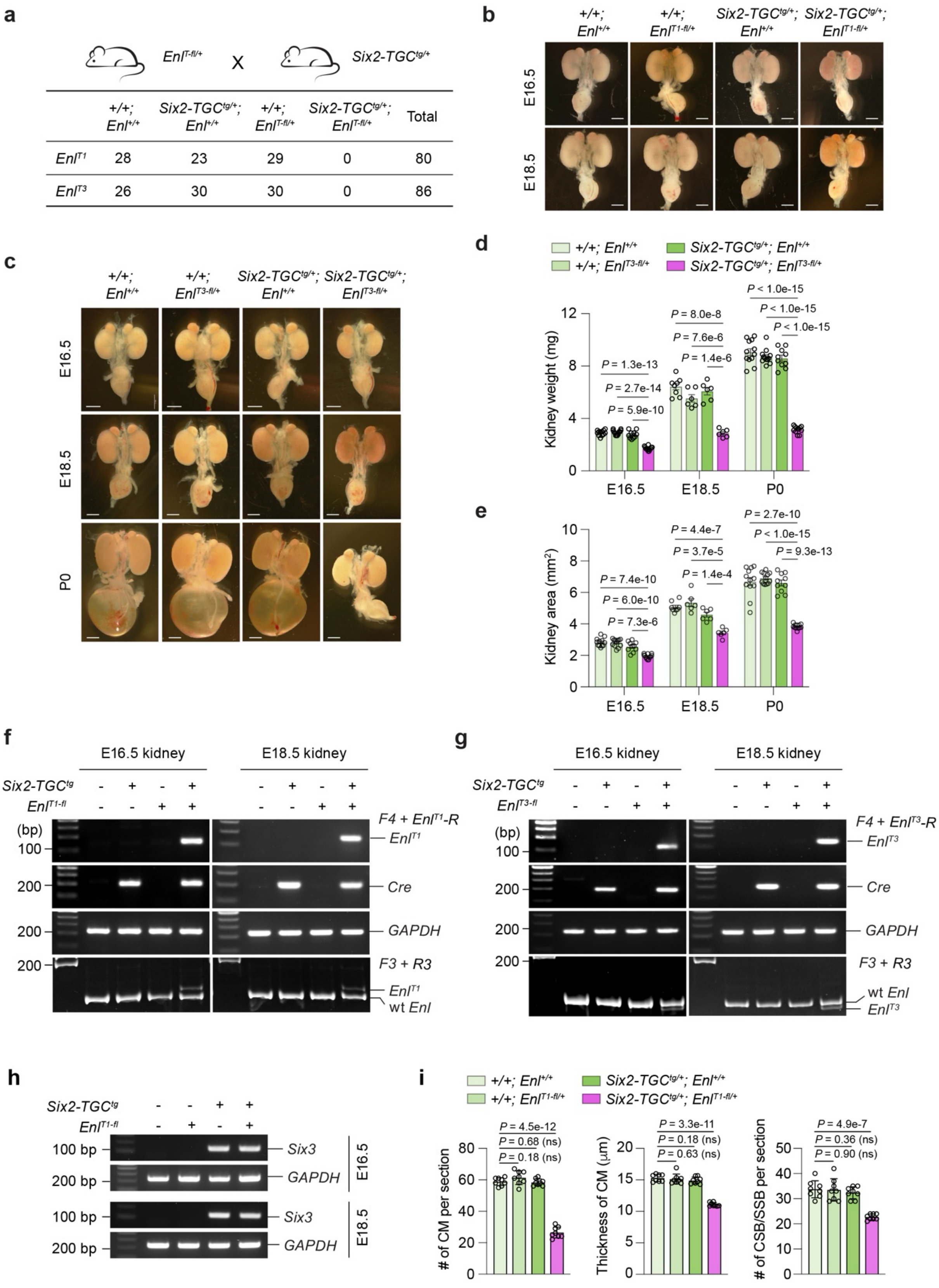

Supplementary Fig. 4 | Kidney developmental defects in Six2-ENL^T mice. a, Neonatal lethality resulting from heterozygous expression of ENL^T mutant driven by *Six2-TGC^{tg}*. Schematic of the breeding strategy is on top. The table lists the numbers of viable progeny with all expected genotypes. **b**, Bright field images of E16.5 (top) and E18.5 (bottom) kidneys with indicated genotypes. Scale bars, 1 mm. **c**, Bright field images of the E16.5 (top), E18.5 (middle) and P0 (bottom) kidneys with indicated genotypes. Scale bars, 1 mm. **d**, **e**, Kidney weight (**d**) and size (**e**) of Six2-ENL^{T3} cohort measured at E16.5, E18.5 and P0. Number of samples at E16.5, E18.5 and P0: *+/+; Enl$^{+/+}$* (*n* = 12, 8, and 12); *+/+; Enl$^{T3\text{-}fl/+}$* (*n* = 12, 6 and 12); *Six2-TGC$^{tg/+}$; Enl$^{+/+}$* (*n* = 10, 6 and 10); and *Six2-TGC$^{tg/+}$; Enl$^{T3\text{-}fl/+}$* (*n* = 12, 6 and 12). **f**, **g**, RT-PCR validation of the expression of wildtype *Enl*, *Enl^T*, *Cre*, and internal control *Gapdh* in Six2-ENL^{T1} (**f**) and Six2-ENL^{T3} (**g**) embryonic kidneys at E16.5 and E18.5. **h**, RT-PCR showing the expression of *Six3* in *Six2-TGC*$^{tg/+}$ kidneys at E16.5 and E18.5. **i**, Quantification of CM and CSB/SSB in E18.5 embryonic kidneys with all expected genotypes from the breeding shown in (**a**). *n* = 8 per genotype. Data in **d**, **e**, and **i** represent mean ± s.d.; two-tailed unpaired Student's *t*-test; ns, not significant. Source data are provided as a Source Data file.

Supplementary Fig. 5

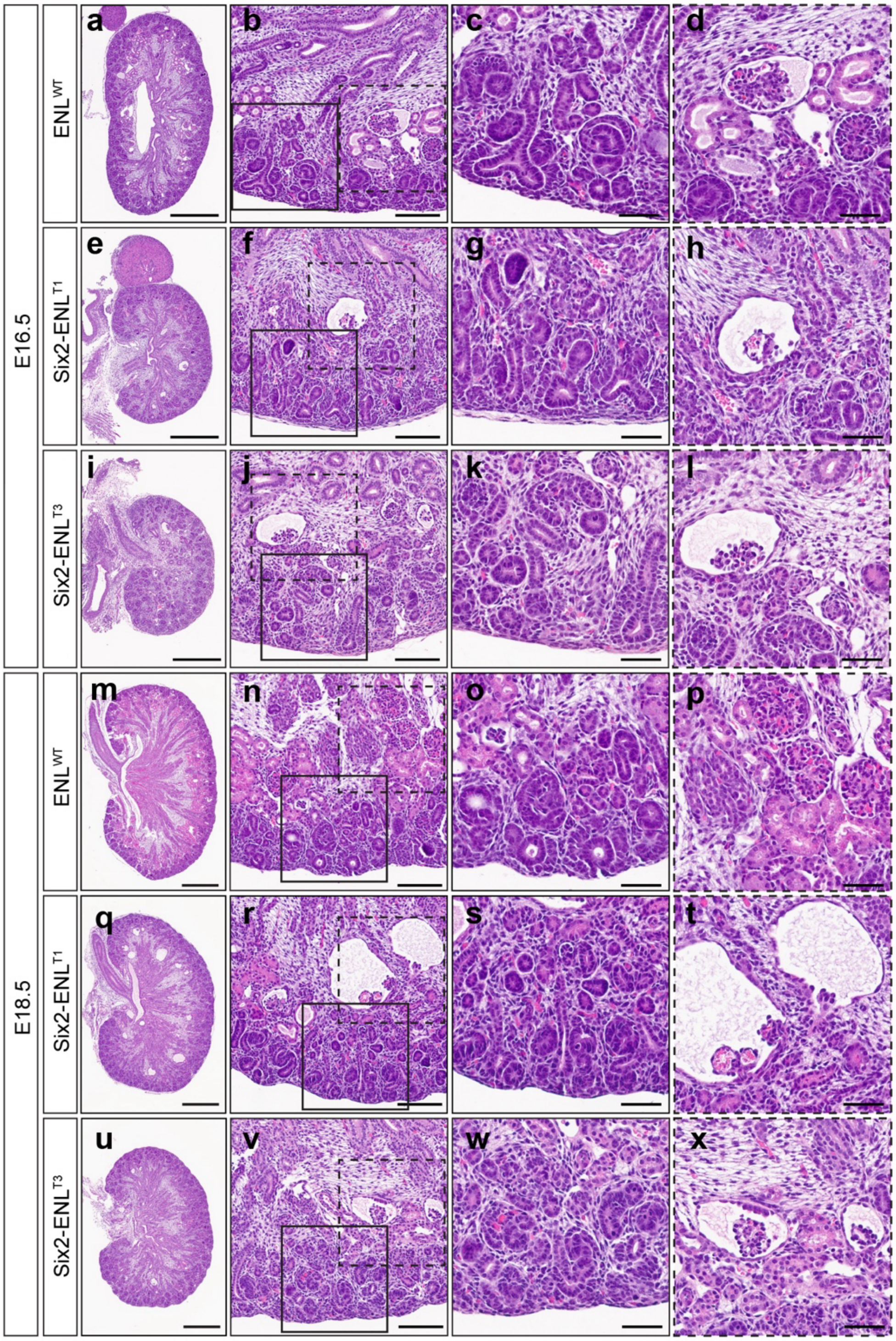

Supplementary Fig. 5 | Histological analysis of nephrogenesis defects in Six2-ENL^{T} mutants at various developmental stages. H&E staining of kidney sections from E16.5 WT (**a**-**d**), Six2-ENL^{T1} (**e**-**h**), and Six2-ENL^{T3} (**i**-**l**); and E18.5 WT (**m**-**p**), Six2-ENL^{T1} (**q**-**t**) and Six2-ENL^{T3} (**u**-**x**) fetuses. Solid line boxes are magnified focusing on nephrogenic zone, and dash line boxes are magnified focusing on glomerulus and renal tubules. Scale bars: 0.5 mm (first column), 0.1 mm (second column), and 50 μm (last two zoom-in columns).

Supplementary Fig. 6

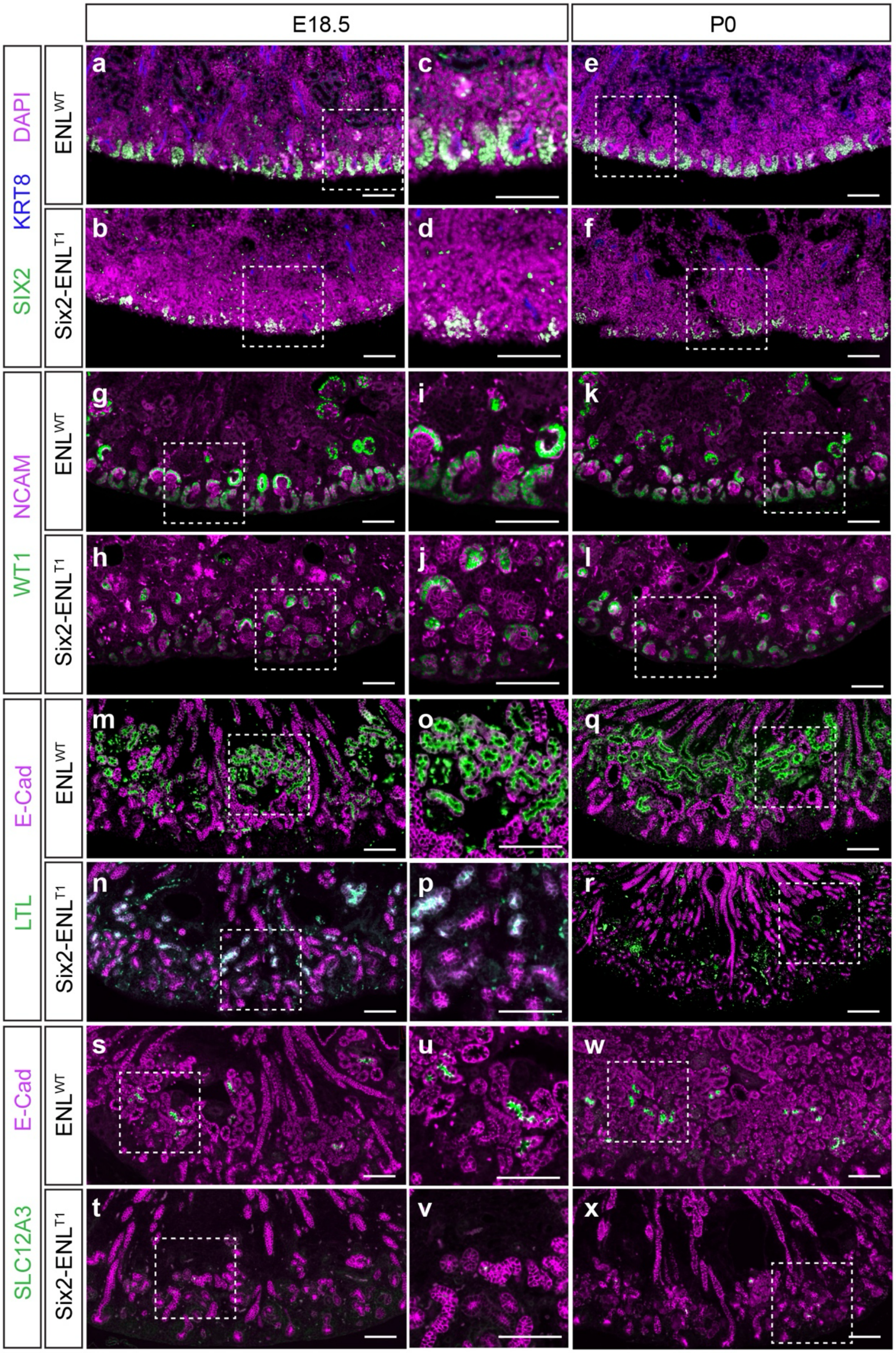

Supplementary Fig. 6 | Immunofluorescence staining of nephron markers in Six2-ENL^{T1} mutant kidneys. Immunofluorescence staining of SIX2 and KRT8 (**a**-**f**), WT1 and NCAM (**g**-**l**), LTL and E-Cad (**m**-**r**), and SLC12A3 and E-Cad (**s**-**x**) in E18.5 and P0 kidney sections from Six2-ENL^{T1} mutants and ENL^{WT} counterparts. Areas framed by dash line boxes in the first column are magnified in the zoomed-in images in the second column. Areas framed by dash line boxes in the third column are magnified in the zoomed-in images shown in **Fig. 2f**-**m**. Scale bars, 100 μm.

Supplementary Fig. 7

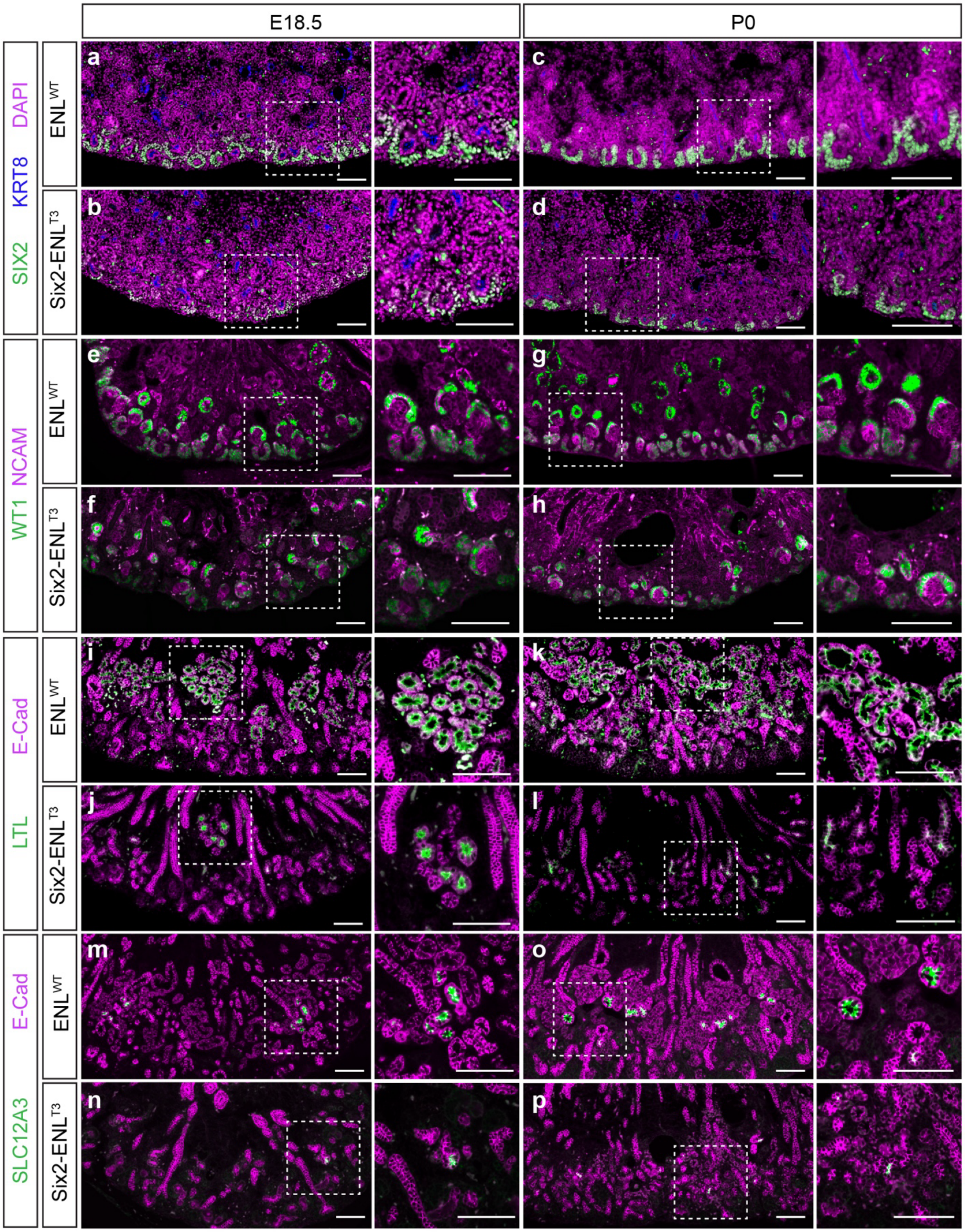

Supplementary Fig. 7 | Immunofluorescence staining of nephron markers in Six2-ENL^{T3} mutant kidneys. Immunofluorescence staining of SIX2 and KRT8 (**a**-**d**), WT1 and NCAM (**e**-**h**), LTL and E-Cad (**i**-**l**), and SLC12A3 and E-Cad (**m**-**p**) in E18.5 and P0 kidney sections from Six2-ENL^{T3} mutants and ENL^{WT} counterparts. Areas framed by dash line boxes in the first and third columns are magnified in the zoomed-in images in the second and fourth columns. Scale bars, 100 μm.

Supplementary Fig. 8

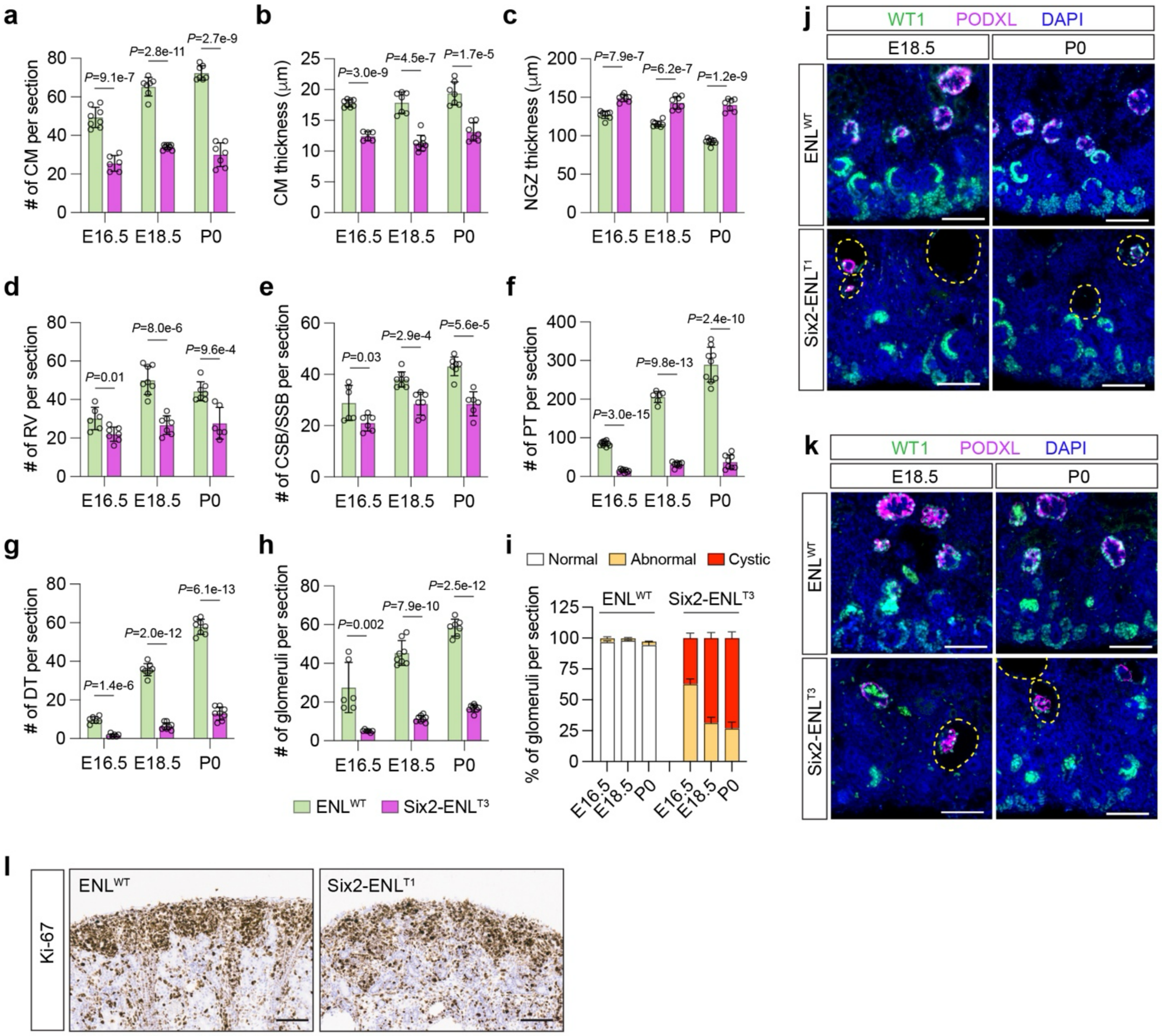

Supplementary Fig. 8 | Quantification of nephrogenesis defects in Six2-ENL^{T3} mutants at various developmental stages. a-**h**, Quantification of nephron structures per section in E16.5, E18.5 and P0 kidneys. CM numbers (**a**) and thickness (**b**) (*n* = 8, 7, 7 ENL^{WT} and 6, 9, 7 ENL^{T3}); NGZ thickness (**c**) (*n* = 8, 8, 8 ENL^{WT} and 7, 8, 7 ENL^{T3}); numbers of RV (**d**) (*n* = 6, 8, 7 ENL^{WT} and 7, 7, 6 ENL^{T3}), CSB-SSB (**e**) (*n* = 6, 8, 7 ENL^{WT} and 6, 7, 6 ENL^{T3}), PT (**f**) (*n* = 10, 6, 9 ENL^{WT} and 8, 8, 8 ENL^{T3}), DT (**g**) (*n* = 6, 8, 8 ENL^{WT} and 6, 8, 8 ENL^{T3}), and glomeruli (**h**) (*n* = 6, 8, 7 ENL^{WT} and 6, 8, 8 ENL^{T3}). **i**, Percentage of glomeruli with normal, abnormal and cystic morphologies in the same set of ENL^{WT} and Six2-ENL^{T3} kidneys as in (**h**). Data represent mean ± s.d.; two-tailed unpaired Student's *t*-test. **j**, **k**, Immunofluorescence staining of WT1 and PODXL in E18.5 and P0 kidney sections from Six2-ENL^{T1} (**j**), Six2-ENL^{T3} (**k**) and the ENL^{WT} counterparts. Scale bars, 100 μm. **l**, Immunohistochemistry staining of Ki-67 in E18.5 ENL^{WT} and Six2-ENL^{T1} kidney sections. Scale bars, 100 μm. For (**j**-**l**), images are representative of three independent experiments. Source data are provided as a Source Data file.

Supplementary Fig. 9

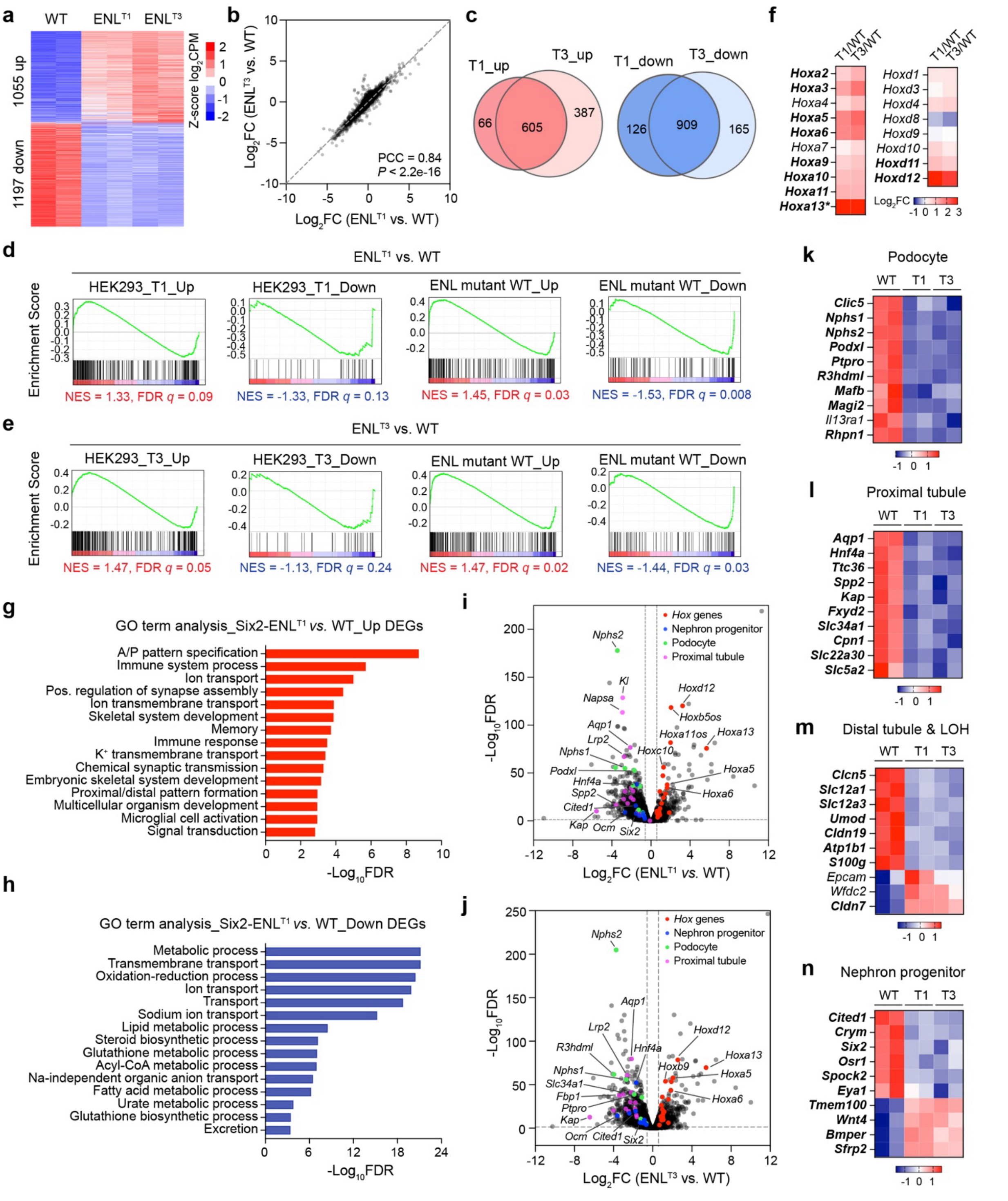

Supplementary Fig. 9 | Global gene expression changes in E18.5 Six2-ENL^{T} embryonic kidneys. a, Heatmap representation of differentially expressed genes (DEGs) in WT, Six2-ENL^{T1} and Six2-ENL^{T3} kidneys at E18.5 (n = 2 per sample group). CPM, counts per million. **b**, Correlation between gene expression changes of Six2-ENL^{T1} *vs.* WT and Six2-ENL^{T3} *vs.* WT. The cumulative distribution function of the *t*-distribution was used for *P*-values in Pearson correlation coefficient. **c**, Venn diagrams showing overlap of genes significantly upregulated and downregulated in Six2-ENL^{T1} and Six2-ENL^{T3} mutant kidneys relative to WT kidneys. **d**, GSEA plots with Six2-ENL^{T1} *vs.* WT as ranking list and curated up and down-regulated genes in HEK293-ENL^{T1} cells and in human Wilms tumors with ENL^{T} mutations as gene sets. **e**, GSEA plots with Six2-ENL^{T3} *vs.* WT as ranking list and curated up and down-regulated genes in HEK293-ENL^{T3} cells and in human Wilms tumors with ENL^{T} mutations as gene sets. NES, normalized enrichment score. In **d** and **e**, positive enrichment scores are highlighted in red, while negative enrichment scores in blue. **f**, Heatmap representations of *Hox* gene expression changes in E18.5 Six2-ENL^{T1} and Six2-ENL^{T3} kidneys relative to WT. DEGs with significant expression change (fold change > 1.5, FDR < 0.05) are highlighted in bold. Color key represents $\log_2$FC of gene expression in ENL^{T} *vs.* WT. $^{*}\log_2$FC of *Hoxa13* is out of range (>3). **g**, **h**, Enriched Gene Ontology (GO) biological process terms of upregulated (**g**) and downregulated (**h**) DEGs in Six2-ENL^{T1}. Top 15 GO terms with FDR < 0.05 are shown. **i**, **j**, Volcano plots of all expressed genes in E18.5 ENL^{T1} (**i**) and ENL^{T3} (**j**) kidneys. The *x*-axis is $\log_2$FC of CPM values from Six2-ENL^{T} *vs.* WT. The y-axis is $-\log_{10}$ transformed FDR values for each gene. *Hox* genes and key marker genes of nephron progenitor, podocyte and proximal tubule are highlighted in red, blue, green and pink, respectively. **k**-**n**, Heatmap representation of marker gene expression for podocyte (**k**), proximal tubule (**l**), distal tubule & loop of Henle (LOH) (**m**), and nephron progenitor (**n**) in WT and ENL^{T} mutant kidneys. DEGs with significant expression change (fold change > 1.5, FDR < 0.05) are highlighted in bold. Color keys represent Z-score $\log_2$CPM.

Supplementary Fig. 10

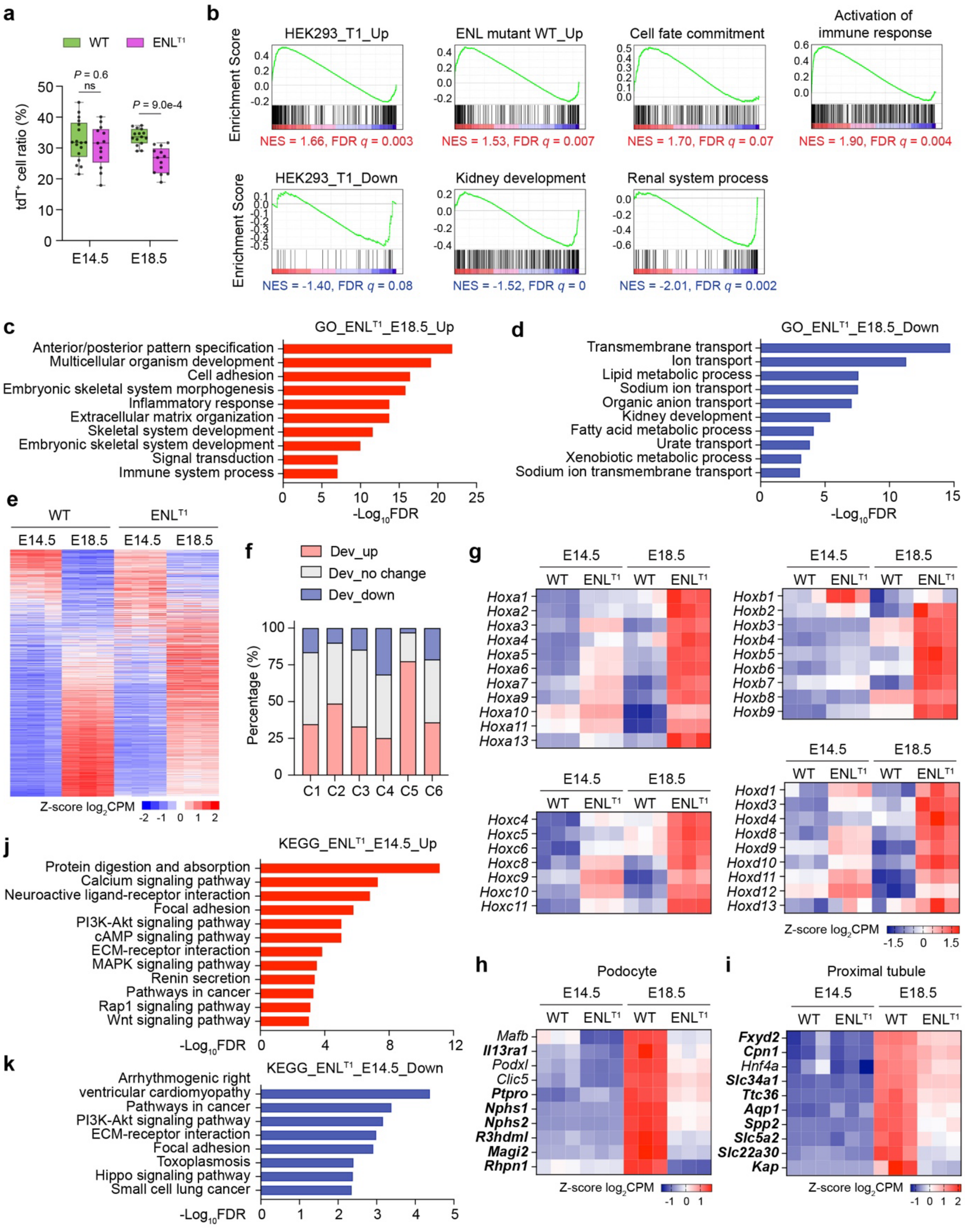

Supplementary Fig. 10 | Gene expression changes in sorted ENL^{T1} mutant cells in Six2-ENL^{T1} kidneys. **a**, Percentage of tdT^{+} cells in WT and Six2-ENL^{T1} kidneys at E14.5 and E18.5 measured by flow cytometry. E14.5: n = 17 WT and 13 ENL^{T1}; E18.5: n = 15 WT and 13 ENL^{T1}. Two-tailed unpaired Student's t-test; ns, not significant. Gating strategy for tdT^{+} cell sorting is included in the Source Data file. **b**, GSEA plots with sorted Six2-ENL^{T1} *vs.* WT kidney cells at E18.5 as ranking list and the indicated curated gene lists and GO terms as gene sets. Positive enrichment scores are highlighted in red, while negative enrichment scores in blue. **c**, **d**, Enriched Gene Ontology (GO) biological process terms of up (**c**) and down (**d**) DEGs in sorted Six2-ENL^{T1} mutant cells at E18.5. Top 10 GO terms with FDR < 0.001 are shown. **e**, Heatmap representation of DEGs identified in sorted Six2-ENL^{T1} cells ranked by their expression changes during development in sorted WT cells (E14.5 *vs.* E18.5). **f**, Distribution of developmentally regulated genes in C1 to C6. **g-i**, Heatmap representations of *Hox* gene (**g**), podocyte (**h**) and proximal tubule (**i**) marker gene expression changes in sorted Six2-ENL^{T1} cells at E14.5 and E18.5. Genes in cluster C5 are highlighted in bold in **h** and **i**. Color keys represent Z-score $\log_2$CPM. **j**, **k**, Enriched KEGG pathways of up (**j**) and down (**k**) DEGs in sorted Six2-ENL^{T1} mutant cells at E14.5. Pathways of up DEGs (FDR < 0.001) and down DEGs (FDR < 0.005) are shown. Source data are provided as a Source Data file.

Supplementary Fig. 11

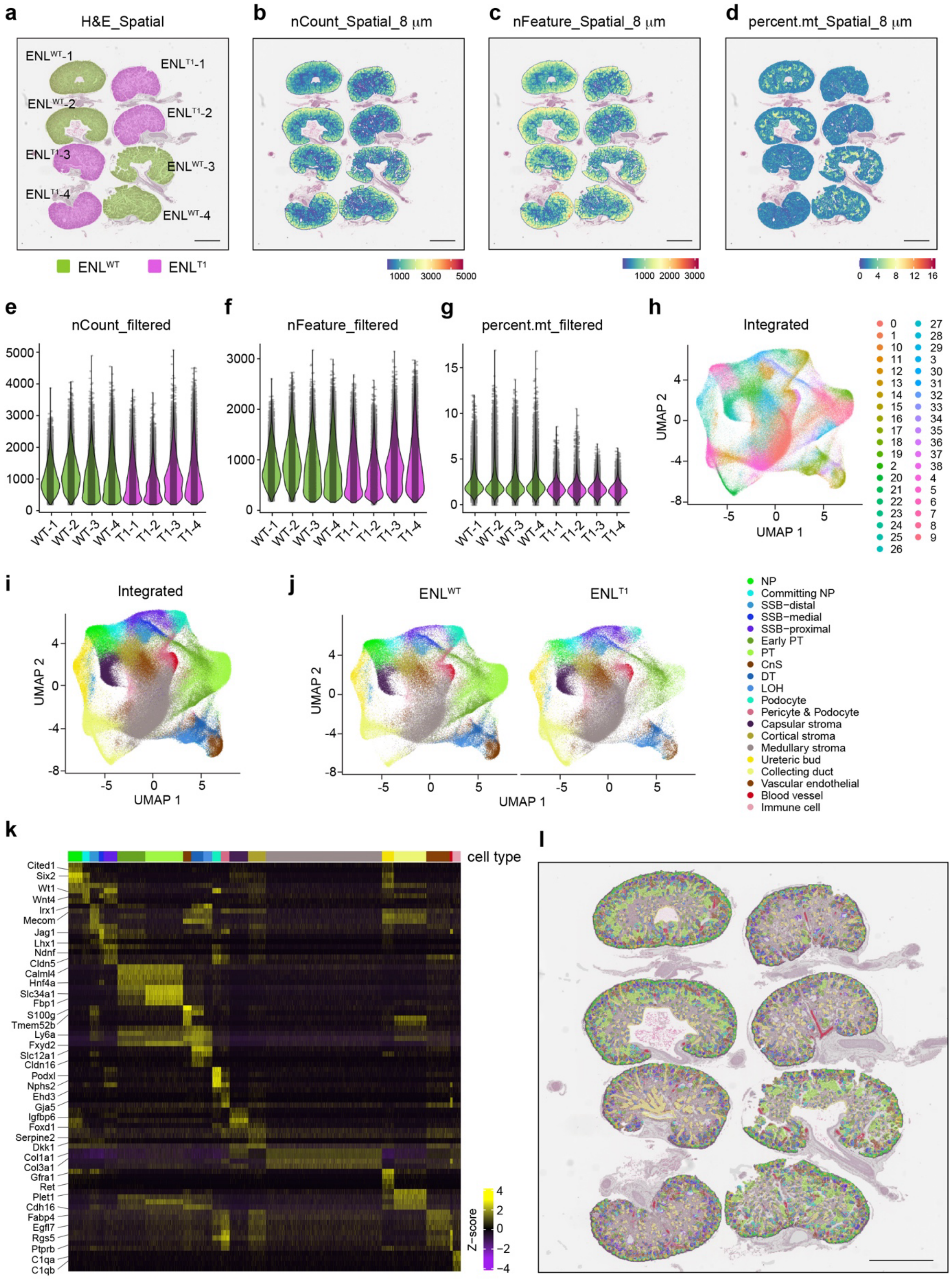

Supplementary Fig. 11 | Visium HD spatial gene expression analysis in Six2-ENL^{T1} kidneys. **a**-**d**, Sample distribution (**a**), 8 µm binned UMI count (**b**), gene count (**c**) and percentage of mitochondrial count (**d**) overlaid on the high-resolution H&E-stained microscope image. Scale bars, 1 mm. **e**-**g**, Violin plots showing the UMI count (**e**), gene count (**f**) and percentage of mitochondrial count (**g**) in 8 µm bins after data filtering. **h**, UMAP projection of integrated 8 µm bins colored by 38 transcriptionally distinct clusters. **i**, **j**, UMAP projection of integrated 8 µm bins (**i**) and bins of WT and ENL^{T1} samples separately (**j**), with bins colored and labeled by annotated cell types. **k**, Heatmap of the expression of key marker genes across cell types as in (**j**). The full gene list is in Supplementary Data 9. **l**, Spatial mapping of eight WT and ENL^{T1} kidney sections with 8 µm bins colored by cell types as in (**j**) within the 6.5 x 6.5 mm capture area. The 20 annotated cell type clusters closely correspond to tissue morphology in the H&E image. Scale bar, 1 mm.

Supplementary Fig. 12

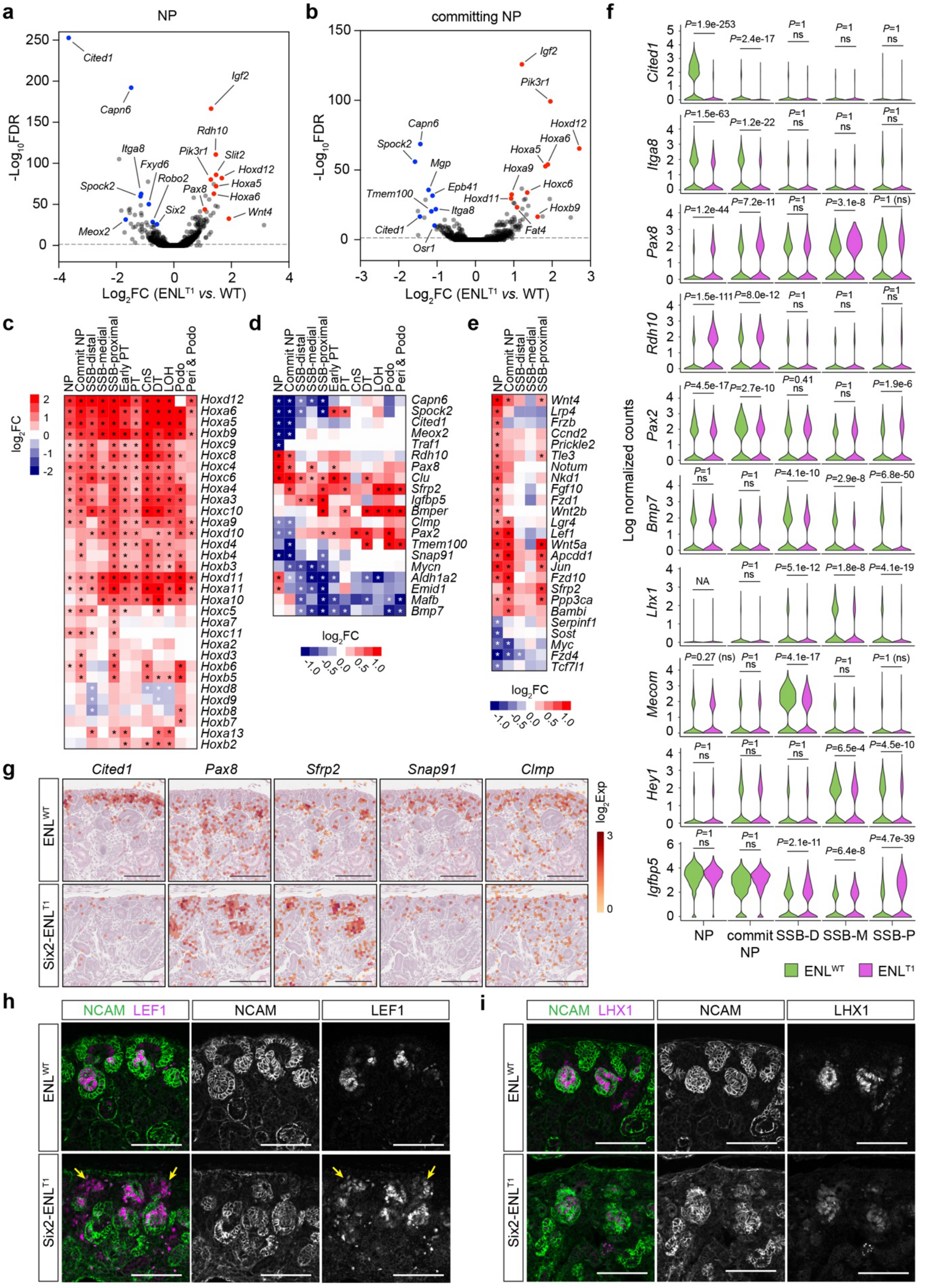

Supplementary Fig. 12 | Dysregulation of NP self-renewal and commitment genes in Six2-ENL^{T1} kidney. **a**, **b**, Volcano plots of all detected genes in NP (**a**) and committing NP (**b**) from spatial transcriptomic analysis. The *x*-axis is $\log_2$FC of gene expression in ENL^{T1} *vs.* WT. The y-axis is -$\log_{10}$ transformed FDR values. Upregulated *Hox* genes and key NP commitment genes in ENL^{T1} are highlighted in red, while downregulated NP "stemness" and commitment genes are highlighted in blue. **c**-**e**, Heatmaps showing expression changes of *Hox* genes (**c**) and key kidney developmental genes (**d**) across nephron cell types, and Wnt signaling pathway genes across NP, committing NP and SSB segments (**e**) in ENL^{T1} mutant. Color keys represent $\log_2$FC of ENL^{T1} *vs.* WT. DEGs with FDR < 0.05 are marked with stars. **f**, Violin plot showing expression levels (log normalized counts) of key nephrogenesis genes in NP, committing NP and SSB segments in WT and ENL^{T1} samples. Wald test with adjusted *P*-values shown; NA, not available; ns, not significant. **g**, Expression and spatial distribution of *Cited1*, *Pax8*, *Sfrp2*, *Snap91*, and *Clmp* in WT and ENL^{T1} kidney sections. Color key represents $\log_2$ transformed UMI counts ($\log_2$Exp) in bins. $n = 4$ WT and 4 ENL^{T1}. Scale bars, 0.1 mm. **h**, **i**, Immunofluorescence staining of NCAM and LEF1 (**h**), and NCAM and LHX1 (**i**) in ENL^{WT} and Six2-ENL^{T1} E18.5 kidney sections. Yellow arrows in **h** indicate abnormal LEF1 activation in the cap mesenchyme and PTA/RV. Scale bars, 100 µm. For (**h**, **i**), images are representative of three independent experiments.

Supplementary Fig. 13

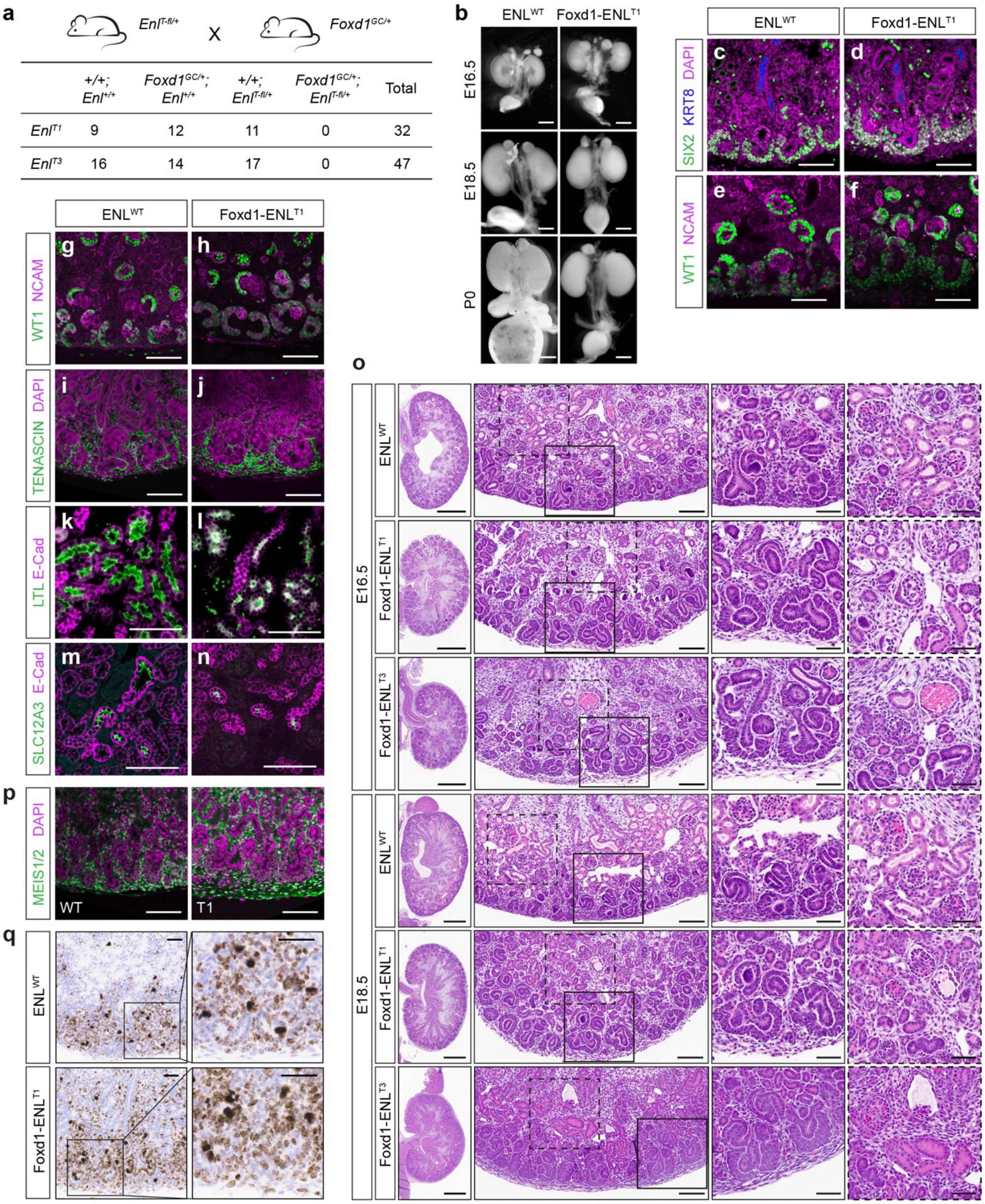

Supplementary Fig. 13 | Kidney developmental defects in Foxd1-ENL^{T} mutant mice. a, Neonatal lethality resulting from heterozygous expression of ENL^{T} mutant driven by *Foxd1^{GC}*. Schematic of the breeding strategy is on top. The table lists the numbers of viable progeny with all expected genotypes. **b**, Bright field images of the E16.5 (top), E18.5 (middle) and P0 (bottom) kidneys with indicated genotypes. Scale bars, 1 mm. **c-f**, Immunofluorescence staining of SIX2 and KRT8 (**c**-**d**), and WT1 and NCAM (**e**-**f**) in E16.5 kidney sections from Foxd1-ENL^{T1} mutants and ENL^{WT} counterparts. **g-n**, Immunofluorescence staining of WT1 and NCAM (**g**-**h**), TENASCIN (**i**-**j**), LTL and E-Cad (**k**-**l**), and SLC12A3 and E-Cad (**m**-**n**) in P0 kidney sections from Foxd1-ENL^{T1} mutants and ENL^{WT} counterparts. Scale bars in **g-n**, 100 μm. **o**, H&E staining of kidney sections from E16.5 and E18.5 ENL^{WT}, Foxd1-ENL^{T1}, and Foxd1-ENL^{T3} fetuses. Solid line boxes are magnified focusing on NGZ, while dash line boxes are magnified focusing on glomeruli and renal tubules. Scale bars: 0.5 mm (first column); 0.1 mm (second column); and 50 μm (last two zoom-in columns). **p,** Immunofluorescence staining of stromal marker MEIS1/2 in E18.5 kidney sections from Foxd1-ENL^{T1} mutants and ENL^{WT} counterparts. Scale bars, 100 μm. **q**, Immunohistochemistry staining of Ki-67 in P0 ENL^{WT} and Foxd1-ENL^{T1} kidney sections. Scale bars, 50 μm. For (**p**, **q**), images are representative of three independent experiments.

Supplementary Fig. 14

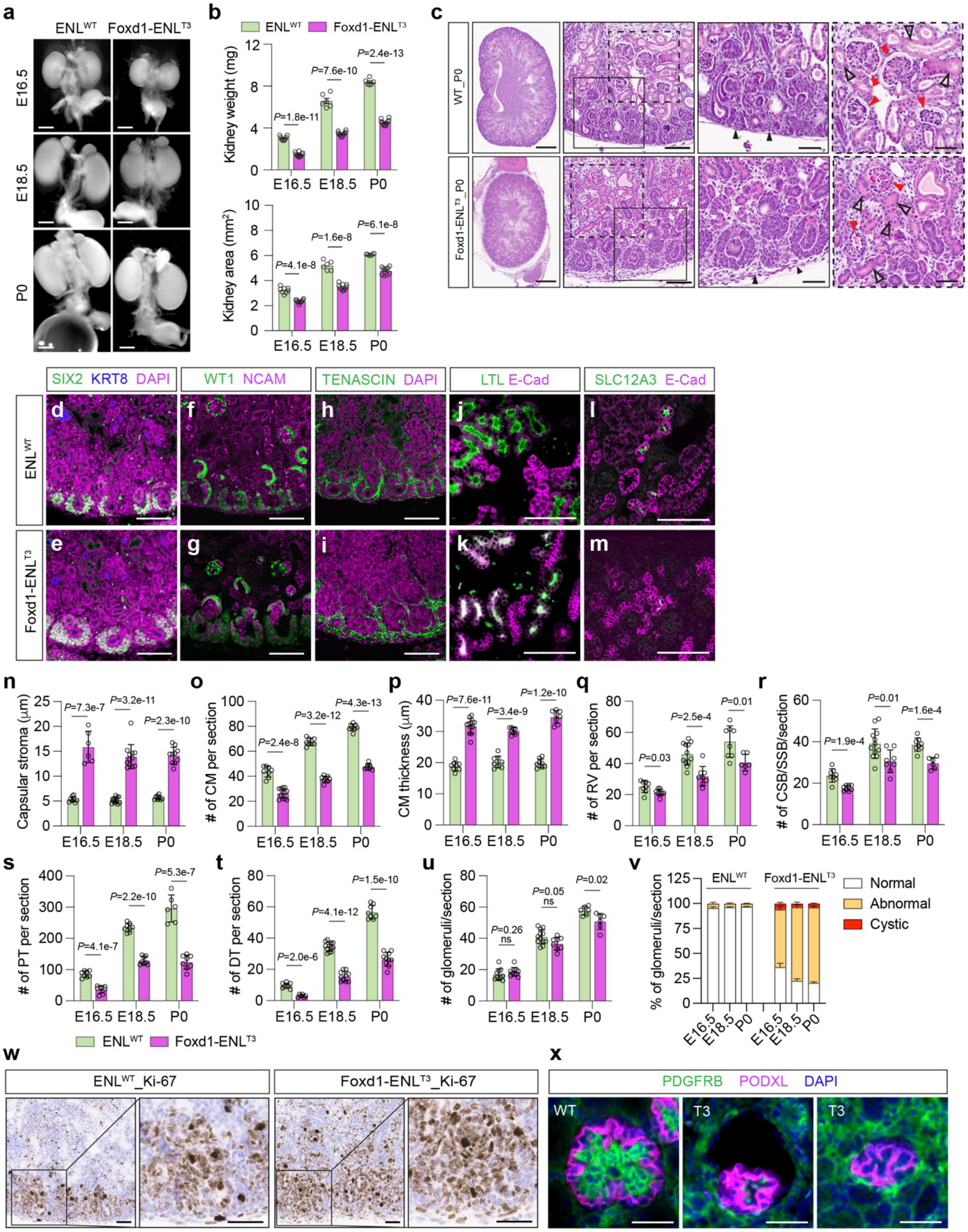

Supplementary Fig. 14 | Quantification of kidney developmental defects in Foxd1-ENL^{T3} mutants at various developmental stages. **a**, Bright field images of the E16.5 (top), E18.5 (middle) and P0 (bottom) kidneys with indicated genotypes. Scale bars, 1 mm. **b**, Kidney weight (top) and size (bottom). Number of samples at E16.5, E18.5 and P0: WT (*+/+; Enl$^{T3-fl/+}$*) (n = 8, 6 and 6); and Foxd1-ENL^{T3} (*Foxd1$^{GC/+}$; Enl$^{T3-fl/+}$*) (n = 10, 10 and 10). Data represent mean ± s.d.; two-tailed unpaired Student's *t*-test. **c**, H&E staining of kidney sections from P0 WT and Foxd1-ENL^{T3} pups. Solid line boxes are magnified focusing on NGZ, while dash line boxes are magnified focusing on glomeruli and renal tubules. Red arrowheads indicate glomerulus; black open triangles indicate proximal tubule; and black triangles indicate capsular stroma, which is thickened in Foxd1-ENL^{T3} mutant. Scale bars, 0.5 mm (first column); 0.1 mm (second column); and 50 μm (last two zoom-in columns). **d**-**m**, Immunofluorescence staining of SIX2 and KRT8 (**d**, **e**), WT1 and NCAM (**f**-**g**), TENASCIN (**h**-**i**), LTL and E-Cad (**j**-**k**), and SLC12A3 and E-Cad (**l**-**m**) in E18.5 kidney sections. Scale bars in **d**-**m**, 100 μm. **n**-**u**, Quantification of nephron structures per section. Capsular stroma thickness (**n**) (*n* = 8, 12, 8 ENL^{WT} and 6, 12, 10 ENL^{T3}); CM number (**o**) and thickness (**p**) (*n* = 8, 8, 8 ENL^{WT} and 12, 8, 8 ENL^{T3}); numbers of RV (**q**) and CSB-SSB (**r**) (*n* = 8, 12, 8 ENL^{WT} and 8, 8, 6 ENL^{T3}), PT (**s**) (*n* = 8, 8, 6 ENL^{WT} and 8, 8, 8 ENL^{T3}), DT (**t**) (*n* = 8, 12, 8 ENL^{WT} and 6, 10, 10 ENL^{T3}) and glomeruli (**u**) (*n* = 10, 12, 6 ENL^{WT} and 10, 8, 6 ENL^{T3}). **v**, Percentage of glomeruli with normal, abnormal and cystic morphologies in the same set of ENL^{WT} and Foxd1-ENL^{T3} kidneys as in (**u**). Data represent mean ± s.d.; two-tailed unpaired *t*-test; ns, not significant. **w**, Immunohistochemistry staining of Ki-67 in P0 ENL^{WT} and ENL^{T3} kidney sections. Scale bars, 50 μm. **x**, Immunofluorescence staining of PDGFRB and PODXL in glomeruli of P0 kidneys. Scale bars, 40 μm. For (**w**, **x**), similar results were obtained in three independent experiments. Source data are provided as a Source Data file.

Supplementary Fig. 15

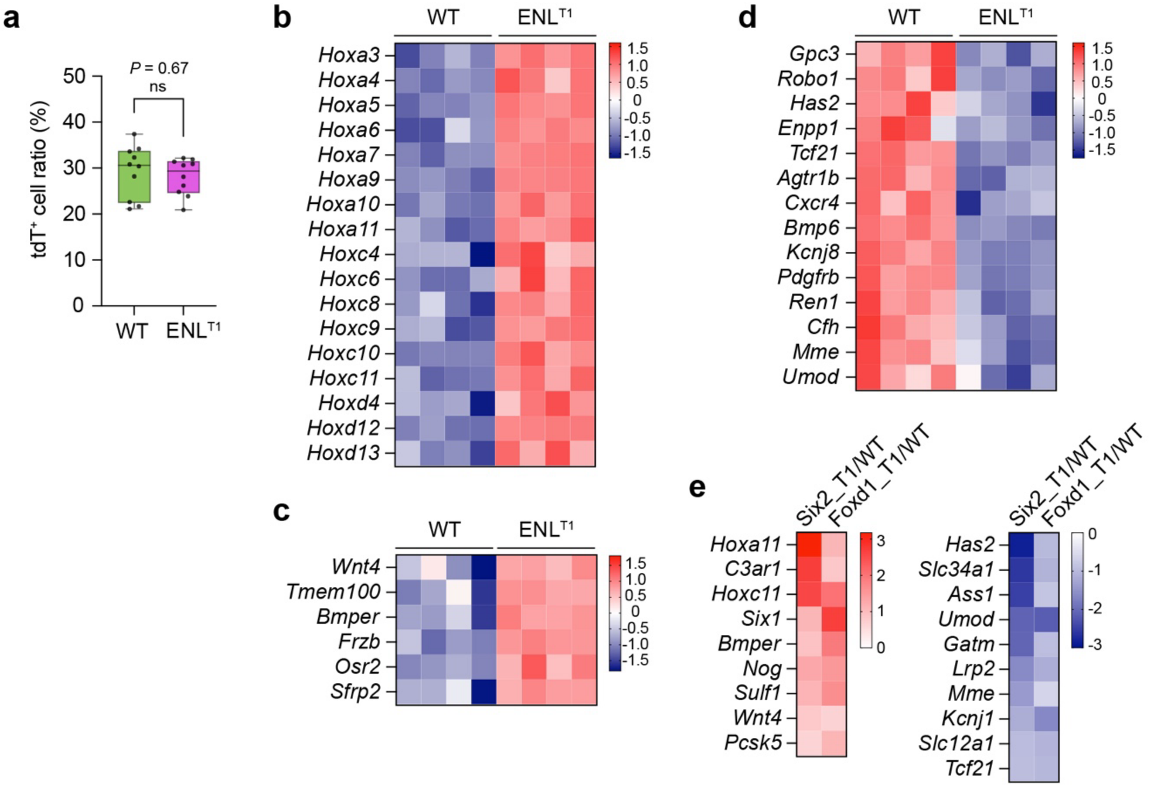

Supplementary Fig. 15 | Gene expression changes in mutant stromal cells of Foxd1-ENL^{T1} kidneys. a, Percentage of tdT^{+} cells in E18.5 WT and Foxd1-ENL^{T1} kidneys measured by flow cytometry. *n* = 10 per sample group. Two-tailed unpaired Student's *t*-test; ns, not significant. Same gating strategy as in Supplementary Fig. 10a was applied. **b-d**, Heatmap depicting upregulation of *Hox* genes (**b**) and progenitor cell commitment and differentiation genes (**c**), along with downregulation of genes associated with the kidney development GO term (**d**) in stromal cells from E18.5 Foxd1-ENL^{T1} kidneys. Color keys in **b-d** represent Z-score $\log_2$CPM. **e**, Heatmap showing expression changes in a common set of DEGs involved in kidney development in both Six2-ENL^{T1} nephron cells and Foxd1-ENL^{T1} stromal cells. Color keys represent $\log_2$FC of ENL^{T1} *vs.* WT. Source data are provided as a Source Data file.

Supplementary Fig. 16

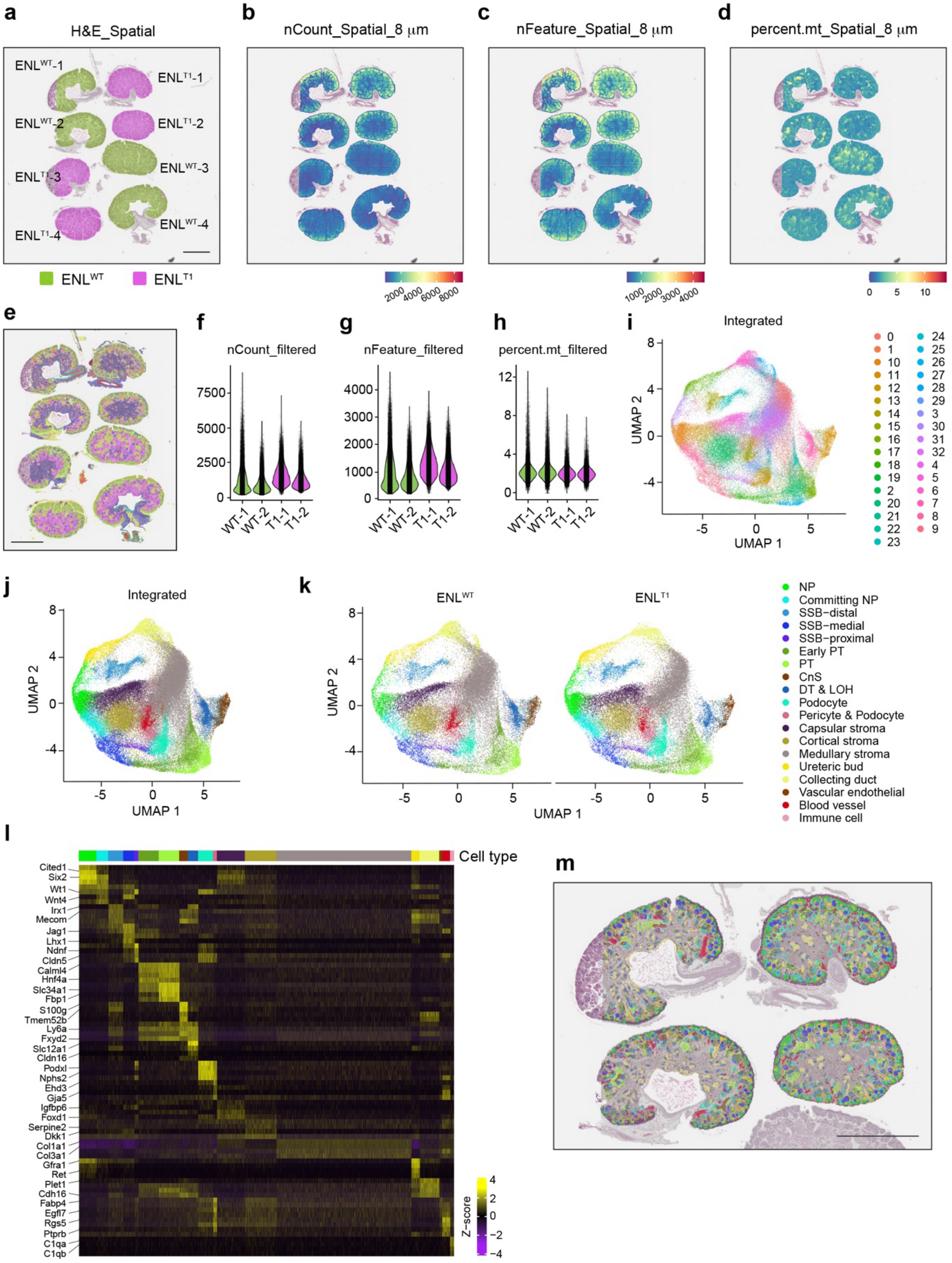

Supplementary Fig. 16 | Visium HD spatial gene expression analysis in Foxd1-ENL^{T1} kidney. a-**d**, Sample distribution (**a**), 8 μm binned UMI count (**b**), gene count (**c**) and percentage of mitochondrial count (**d**) overlaid on the high-resolution H&E-stained microscope image. The UMI count distribution patterns in the bottom four samples differ from the top four samples and the SIX2 spatial transcriptomic dataset (Supplementary Fig. 11b). **e**, Graph-based clustering reveals distinct patterns between the top four and bottom four kidney samples within the 6.5 x 6.5 mm capture area. Scale bars in **a**-**e**, 1 mm. **f**-**h**, Violin plots showing the UMI count (**f**), gene count (**g**) and percentage of mitochondrial count (**h**) in 8 μm bins after sample removal and data filtering. **i**, UMAP projection of integrated 8 μm bins colored by 32 transcriptionally distinct clusters. **j**, **k**, UMAP projection of integrated 8 μm bins (**j**) and bins of WT and ENL^{T1} samples separately (**k**), with bins colored and labeled by annotated cell types. **l**, Heatmap of key marker gene expression across cell types as in (**k**). The full gene list is in Supplementary Data 9. **m**, Spatial mapping of four WT and ENL^{T1} kidney samples with 8 μm bins colored by cell types as in (**k**). The 19 annotated cell type clusters closely correspond to tissue morphology in the H&E image. Scale bar, 1 mm.

Supplementary Fig. 17

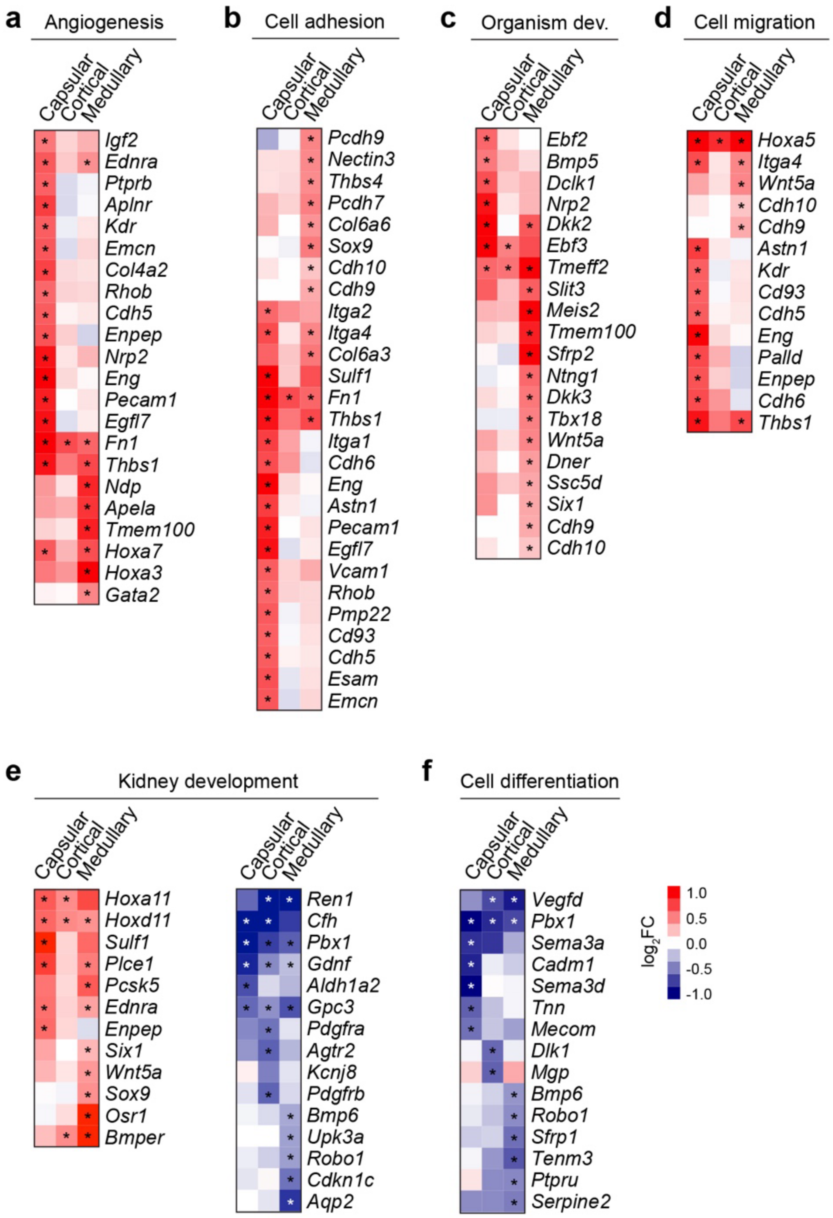

Supplementary Fig. 17 | Gene expression changes in stroma of Foxd1-ENL^{T1} mutant by spatial transcriptomic analysis. a-f, Heatmap representation of expression changes across capsular, cortical and medullary stroma clusters in Foxd1-ENL^{T1} samples for genes involved in angiogenesis (**a**), cell adhesion (**b**), multicellular organism development (**c**), cell migration (**d**), kidney development (**e**), and cell differentiation (**f**). The color key represents log_2FC of ENL^{T1} *vs.* WT. DEGs with FDR < 0.05 are marked with stars.

Supplementary Fig. 18

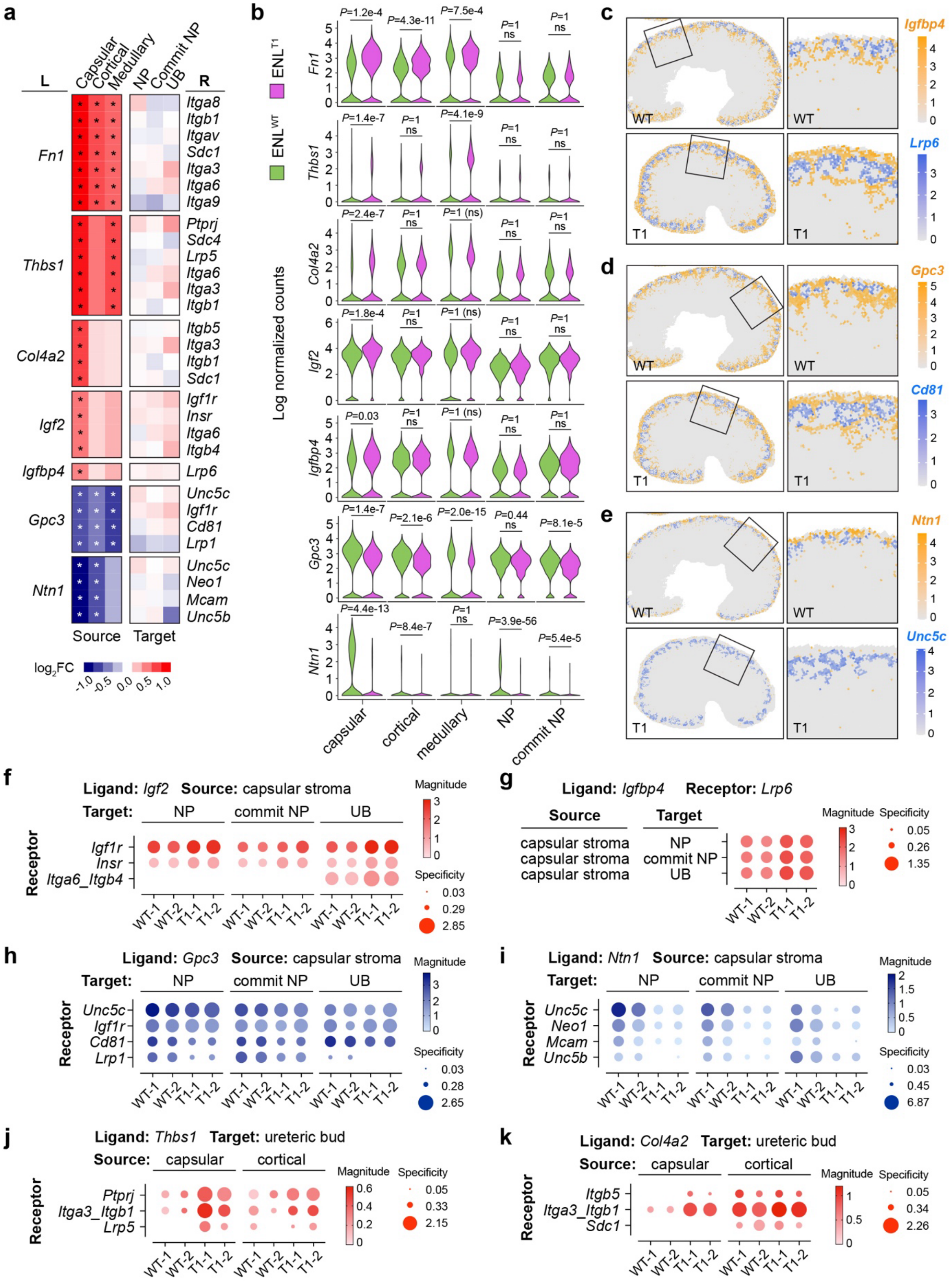

Supplementary Fig. 18 | Spatial transcriptomic analysis reveals changes in paracrine ligand-receptor interactions in Foxd1-ENL^{T1} kidneys. a, Heatmap showing expression changes of ligand genes in stroma clusters and their corresponding receptor genes in NP, committing NP and UB clusters in Foxd1-ENL^{T1} samples. L, ligand; R, receptor. Color key represents log_2FC of ENL^{T1} *vs.* WT. DEGs with FDR < 0.05 are marked with stars. **b**, Violin plots showing expression levels (log normalized counts) of ligand genes *Fn1*, *Thbs1*, *Col4a2*, *Igf2*, *Igfbp4*, *Gpc3*, and *Ntn1* in the stroma, NP, and committing NP of WT and ENL^{T1} samples. Wald test with adjusted *P*-values shown; ns, not significant. **c**-**e**, Spatial expression and distribution of ligand-receptor gene pairs *Igfbp4-Lrp6, Gpc3-Cd81*, and *Ntn1-Unc5c* in the nephrogenic zone stroma and NP/committing NP, respectively. Color key represents log normalized counts. **f**-**k**, Bubble plots depicting the strength and specificity of paracrine ligand-receptor interactions between stromal ligands *Igf2* (**f**), *Igfbp4* (**g**), *Gpc3* (**h**), *Ntn1* (**i**), Thbs1 (**j**) and *Col4a2* (**k**) and their engaged receptors in NP, committing NP or UB in WT and ENL^{T1} samples. Upregulated ligands are indicated in red, while downregulated ligands are shown in blue. Color key represents log magnitude *P*-values. Bubble size denotes log specificity *P*-values. Robust Rank Aggregation was used for *P*-value calculation.
