# Supplementary material for: Expression of ENL YEATS domain tumor mutations in nephrogenic or stromal lineage impairs kidney development
Source: Nat Commun. 2025 Mar 14;16:2531. doi: 10.1038/s41467-025-57926-z (PMC11909213; doi:10.1038/s41467-025-57926-z)
Supplement: Supplementary file 2 — Description of Additional Supplementary Files [file 41467_2025_57926_MOESM2_ESM.pdf]

Description of Additional Supplementary Files

Supplementary Data 1. Differentially expressed genes (DEGs) identified in E18.5 Six2-ENL^{T} kidneys

Supplementary Data 2. DEGs identified in human Wilms tumor with versus without ENL mutations in TARGET dataset

Supplementary Data 3. Gene ontology (GO) analysis of DEGs identified in E18.5 Six2-ENL^{T} kidneys

Supplementary Data 4. DEGs identified in sorted tdT^{+} Six2-ENL^{T1} mutant cells from E14.5 and E18.5 kidneys

Supplementary Data 5. GO analysis of DEGs identified in sorted tdT^{+} Six2-ENL^{T1} mutant cells from E14.5 and E18.5 kidneys

Supplementary Data 6. Grouped DEGs identified in sorted tdT^{+} Six2-ENL^{T1} mutant cells from E14.5 and E18.5 kidneys

Supplementary Data 7. GO analysis of DEG clusters from sorted tdT^{+} Six2-ENL^{T1} mutant cells in E14.5 and E18.5 kidneys

Supplementary Data 8. KEGG analysis of DEGs identified in sorted tdT^{+} Six2-ENL^{T1} mutant cells from E14.5 and E18.5 kidneys

Supplementary Data 9. Cell type marker genes in embryonic kidney spatial transcriptomic analysis

Supplementary Data 10. GO analysis of DEGs identified in spatial transcriptomic analysis of Six2-ENL^{T1} kidneys

Supplementary Data 11. DEGs identified in spatial transcriptomic analysis of Six2-ENL^{T1} kidneys

Supplementary Data 12. DEGs identified in sorted tdT^{+} Foxd1-ENL^{T1} mutant cells from E18.5 kidneys

Supplementary Data 13. GO analysis of DEGs identified in sorted tdT^{+} Foxd1-ENL^{T1} mutant cells from E18.5 kidneys

Supplementary Data 14. Comparison of DEGs identified in sorted tdT^{+} Six2-ENL^{T1} and Foxd1-ENL^{T1} mutant cells

Supplementary Data 15. DEGs identified in spatial transcriptomic analysis of Foxd1-ENL^{T1} kidneys

Supplementary Data 16. GO analysis of DEGs identified in spatial transcriptomic analysis of Foxd1-ENL^{T1} kidneys

Supplementary Data 17. Oligos used in this study

Supplementary Data 18. Antibodies used in this study

Supplementary Data 19. Gene sets used in GSEA analysis in this study
