# Supplementary material for: Expression of ENL YEATS domain tumor mutations in nephrogenic or stromal lineage impairs kidney development
Source: Nat Commun. 2025 Mar 14;16:2531. doi: 10.1038/s41467-025-57926-z (PMC11909213; doi:10.1038/s41467-025-57926-z)
Supplement: Supplementary file 4 — Reporting Summary [file 41467_2025_57926_MOESM4_ESM.pdf]

nature portfolio

Corresponding author(s): Hong Wen

Last updated by author(s): Feb 20, 2025

Reporting Summary

Nature Portfolio wishes to improve the reproducibility of the work that we publish. This form provides structure for consistency and transparency in reporting. For further information on Nature Portfolio policies, see our Editorial Policies and the Editorial Policy Checklist.

Statistics

For all statistical analyses, confirm that the following items are present in the figure legend, table legend, main text, or Methods section.

n/a	Confirmed	
☐	☒	The exact sample size (*n*) for each experimental group/condition, given as a discrete number and unit of measurement
☐	☒	A statement on whether measurements were taken from distinct samples or whether the same sample was measured repeatedly
☐	☒	The statistical test(s) used AND whether they are one- or two-sided<br>*Only common tests should be described solely by name; describe more complex techniques in the Methods section.*
☐	☒	A description of all covariates tested
☒	☐	A description of any assumptions or corrections, such as tests of normality and adjustment for multiple comparisons
☐	☒	A full description of the statistical parameters including central tendency (e.g. means) or other basic estimates (e.g. regression coefficient) AND variation (e.g. standard deviation) or associated estimates of uncertainty (e.g. confidence intervals)
☐	☒	For null hypothesis testing, the test statistic (e.g. *F*, *t*, *r*) with confidence intervals, effect sizes, degrees of freedom and *P* value noted<br>*Give P values as exact values whenever suitable.*
☒	☐	For Bayesian analysis, information on the choice of priors and Markov chain Monte Carlo settings
☒	☐	For hierarchical and complex designs, identification of the appropriate level for tests and full reporting of outcomes
☐	☒	Estimates of effect sizes (e.g. Cohen's *d*, Pearson's *r*), indicating how they were calculated

Our web collection on statistics for biologists contains articles on many of the points above.

Software and code

Policy information about availability of computer code

Data collection

Illumina NovaSeq 6000 was used to collect RNA-seq data.
10X GENOMICS Visium HD platform, Visium CytAssist, and Element AVITI sequencer were used to collect spatial transcriptomics data.
Zeiss Discovery V12 microscope for bright field kidney imaging.
Leica Aperio AT2 scanner for H&E and IHC image scanning.
Zeiss Axioscan 7 scanner and Nikon A1 plus-RSi laser scanning confocal microscope for immunofluorescence imaging.
BD FACSymphony S6 was used to sort tdTomato+ cells from kidneys.

Data analysis

For bulk RNA-seq:
Base calling was done by Illumina RTA3 and output of NCS was demultiplexed and converted to FastQ format with Illumina Bcl2fastq.
Fastq files were mapped to mm10 mouse genome by HISAT2 (v2.1.0).
Gene reads count tables were generated by HTSeq (v0.11.3).
CPM (counts per million) and fold change values were calculated edgeR (v3.16.5).
For spatial RNA-seq:
Base calling was performed on instrument and AVITI OS (v2.6.2).
Output was demultiplexed and converted to FastQ format with Element Biosciences Bases2fastq (v1.7.0).
Fastq files were mapped to mm10 mouse genome by SpaceRanger (v3.0.1).
Annotation of bins to samples based on overlaying bins on the microscope images was done with Loupe Browser v8.0.0.
Quality control of bins, batch correction, PCA, UMAP, clustering, differential expression testing and pathway analyses were done in R v4.4.0

using the packages, Seurat v5.1.0, metap v1.11, DESeq2 v1.45.3, clusterProfiler v4.13.0, enrichplot v1.25.0, dittoSeq v1.17.0 and ComplexHeatmap v2.21.1.
Ligand and receptor coexpression analyses were done in Python v3.10.0 using the packages, LIANA+ v1.4.0 and ScanPy v1.10.4.
Aperio eSlide Manager, Fiji/ImageJ2 v2.14.0 and QuPath v0.5.1 were used for image analysis.
BD FACSDiva and FlowJo v10.10.0 were used for flow cytometry.
GraphPad Prism 10 was used for generating graphs and plots.

For manuscripts utilizing custom algorithms or software that are central to the research but not yet described in published literature, software must be made available to editors and reviewers. We strongly encourage code deposition in a community repository (e.g. GitHub). See the Nature Portfolio guidelines for submitting code & software for further information.

Data

Policy information about availability of data

All manuscripts must include a data availability statement. This statement should provide the following information, where applicable:

- Accession codes, unique identifiers, or web links for publicly available datasets
- A description of any restrictions on data availability
- For clinical datasets or third party data, please ensure that the statement adheres to our policy

The authors declare that raw data supporting the findings of this study are included in this published artical and its Supplementary Information files. Bulk RNA-seq and spatial transcriptomic data described in the manuscript have been deposited in the NCBI Gene Expression Omnibus (GEO) database under accession numbers GSE266256 and GSE283433, respectively. Source data are provided with this paper.

Research involving human participants, their data, or biological material

Policy information about studies with human participants or human data. See also policy information about sex, gender (identity/presentation), and sexual orientation and race, ethnicity and racism.

Reporting on sex and gender	Not applicable.
Reporting on race, ethnicity, or other socially relevant groupings	Not applicable.
Population characteristics	Not applicable.
Recruitment	Not applicable.
Ethics oversight	Not applicable.

Note that full information on the approval of the study protocol must also be provided in the manuscript.

Field-specific reporting

Please select the one below that is the best fit for your research. If you are not sure, read the appropriate sections before making your selection.

[x] Life sciences [ ] Behavioural & social sciences [ ] Ecological, evolutionary & environmental sciences

For a reference copy of the document with all sections, see nature.com/documents/nr-reporting-summary-flat.pdf

Life sciences study design

All studies must disclose on these points even when the disclosure is negative.

Sample size	No statistical method was used to predetermine sample size. Sample size was determined according to standard practices and was sufficient for statistical analysis. For morphology and quantification of nephron structures in kidneys, >= 5 samples per sample group per time point were analyzed. Exact numbers are included in corresponding figure legends. Three biological replicates were used in RT-PCR. Two to three biological replicates were used in whole kidney RNA-seq. Three to four biological replicates were used in sorted tdT+ cell RNA-seq. Four biological replicates were included in Visium HD spatial gene expression analysis.
Data exclusions	Four kidney sections (WT-3, WT-4, T1-3, and T1-4) were excluded in the final analysis of FOXD1 Visium HD spatial gene expression dataset. They display different spatial distribution of UMI count numbers and graph-based clustering patterns compared with other samples in the same dataset and the SIX2 dataset.Raw data of these four samples are included in the GEO submission and available.
Replication	The number of biological replicates was indicated in corresponding figure legend.

Randomization	Randomization was not applicable in this study. Enl T-fl mice were crossed with Cre mouse stains, mice with all expected genotypes were used in this study.
Blinding	Blinding was not applicable, since samples were grouped by genotypes. To ensure consistency, control and experimental samples were always processed in parallel with identical protocols.

Reporting for specific materials, systems and methods

We require information from authors about some types of materials, experimental systems and methods used in many studies. Here, indicate whether each material, system or method listed is relevant to your study. If you are not sure if a list item applies to your research, read the appropriate section before selecting a response.

Materials & experimental systems

n/a	Involved in the study	
☐	☒	Antibodies
☐	☒	Eukaryotic cell lines
☒	☐	Palaeontology and archaeology
☐	☒	Animals and other organisms
☒	☐	Clinical data
☒	☐	Dual use research of concern
☒	☐	Plants

Methods

n/a	Involved in the study	
☒	☐	ChIP-seq
☐	☒	Flow cytometry
☒	☐	MRI-based neuroimaging

Antibodies

Antibodies used

Target,Supplier,Catalog No., clone name, lot number, Application/ Dilution
Rabbit polyclonal anti-Six2, MyBioSource, MBS7604120, 20201118, IF/1:200
Rabbit monoclonal anti-WT1, abcam, ab89901 , clone CAN-R9(IHC)-56-2, GR3270281-1, IF and IHC/1:300
Fluorescein-LTL, Vector Laboratories, F1321, ZK1006, IF/1:200
Mouse monoclonal anti-KRT8, DSHB, TROMA-I-S, IF/1:50
Mouse monoclonal anti-Ecad, BD Biosciences, 610181, clone 36 ,8274692, IF and IHC/1:200
Mouse monoclonal anti-PODXL, R&D systems, MAB1556, clone 192703 ,IPF0320031, IF/1:200
Rabbit monoclonal anti-PDGFR, abcam, ab32570 , clone Y92, 1042141-19, IF/1:100
Rabbit monoclonal anti-Ki-67, abcam, ab16667 , clone SP6, GR3313195-18, IHC/1:100
Rabbit polyclonal anti-Tenascin, Sigma, AB19011, 3991492, IF/1:100
Rabbit polyclonal anti-Slc12a3, Abcam, ab95302, 1063207-1, IF/1:100
Mouse monoclonal anti-NCAM, Sigma, C9672, clone NCAM-0B11 ,058M4809V, IF/1:50
Rabbit monoclonal anti-Jag1, CST, 2620S , clone 28H8, lot 9, IF/1:50
Rabbit monoclonal anti-LEF1, CST, 2230S , clone C12A5, lot 8, IF/1:250
Rabbit polyclonal anti-LHX1, Abcam, ab229474, 1010023-6, IF/1:100
Mouse monoclonal anti-MEIS1/2/3, Active Motif, #39795, clone 9.2.7, 24010141-11, IF/1:100
Rabbit polyclonal anti-FN1, Proteintech, #15613-1-AP, 00159837, IF/1:200
Goat polyclonal anti-ITGA8, R&D Systems, AF4076-SP, YZZ03231111, IF/1:100
Alexa Flour 488 goat anti-rabbit, Invitrogen, A11034, 2069632, IF/1:500
Alexa Flour 488 goat anti-mouse, Invitrogen, A11001, 2821059, IF/1:500
Alexa Flour 488 donkey anti-goat, Invitrogen, A11055, IF/1:500
Alexa Flour 546 goat anti-rabbit, Invitrogen, A11035, 2701068, IF/1:500
Alexa Flour 546 goat anti-mouse, Invitrogen, A11030, 1345046, IF/1:500
Alexa Flour 546 goat anti-rat, Invitrogen, A11081, 870921, IF/1:500

Validation

All these antibodies were commercially obtained and validated by vendors and multiple published studies, see manufacture's website for references.
Target,Supplier,Catalog No, species, application, validation statements, manufacture's website
Rabbit polyclonal anti-Six2, MyBioSource, MBS7604120, human/mouse, WB/IF, https://www.mybiosource.com/polyclonal-human-mouse-rat-antibody/six2/7604120
Rabbit monoclonal anti-WT1, abcam, ab89901 , human/mouse, WB/IF/IHC, RRID:AB_2043201, https://www.abcam.com/en-us/products/primary-antibodies/wilms-tumor-protein-antibody-can-r9ihc-56-2-ab89901
Fluorescein-LTL, Vector Laboratories, F1321, mouse, IF, RRID:AB_2336560, https://vectorlabs.com/products/fluorescein-lotus-tetragonolobus-lectin-ltl?srsltid=AfmBOoq6g5paeq501eyghZfquuY933_AZAsa_KV9ro1YPqg3Du1MJqh1
Mouse monoclonal anti-KRT8, DSHB, TROMA-I-S, human/mouse/canine, WB/IF/FACS, https://vectorlabs.com/products/fluorescein-lotus-tetragonolobus-lectin-ltl?srsltid=AfmBOoq6g5paeq501eyghZfquuY933_AZAsa_KV9ro1YPqg3Du1MJqh1
Mouse monoclonal anti-Ecad, BD Biosciences, 610181, human/mouse/rat/dog, WB/IF/IHC, https://www.bdbiosciences.com/en-ie/products/reagents/microscopy-imaging-reagents/immunofluorescence-reagents/purified-mouse-anti-e-cadherin.610181
Mouse monoclonal anti-PODXL, R&D systems, MAB1556, mouse, WB/IF, https://www.rndsystems.com/products/mouse-

podocalyxin-antibody-192703_mab1556?gad_source=1&gbraid=0AAAAAD_kmX2nzt33VEZXAIzWYa7pEkxj5&gclid=CjwKCAiA9IC6BhA3EiwAsbltOIBy3-3d80znnoDew1r4B57VDJuTasy46VWxNOR0sq1LZaixzH7McxoC5oQQAvD_BwE&gclsrc=aw.ds
Rabbit monoclonal anti-PDGFR, abcam, ab32570, human/mouse/rat, WB/IF/IHC/FACS, RRID:AB_777165, https://www.abcam.com/en-us/products/primary-antibodies/pdgfr-alpha-pdgfr-beta-antibody-y92-c-terminal-ab32570#application=icc-if
Rabbit monoclonal anti-Ki-67, abcam, ab16667, human/mouse/rat, WB/IF/IHC/FACS, RRID:AB_302459, https://www.abcam.com/en-us/products/primary-antibodies/ki67-antibody-sp6-ab16667
Rabbit polyclonal anti-Tenascin, Sigma, AB19011, human/mouse, WB/IF/IHC, RRID:AB_2203804, https://www.sigmaaldrich.com/US/en/product/mm/ab19011?srsltid=AfmBOopsmNvDEMOqAmwusnI2cJPNbOIyJFoTK0JQLmIp-1OXSpTIbwF3
Rabbit polyclonal anti-Slc12a3, Abcam, ab95302, human/mouse/rat, WB/IF/IHC, RRID:AB_10673907, https://www.abcam.com/en-us/products/primary-antibodies/slc12a3-antibody-ab95302?srsltid=AfmBOopfnOax4z096NI4kYYhUq1P6uO4bHPqiztlMBwMkziY-ft4ocvJ
Mouse monoclonal anti-NCAM, Sigma, C9672, human/mouse/rat, WB/IF/IHC, RRID:AB_1079450, https://www.sigmaaldrich.com/US/en/product/sigma/c9672?srsltid=AfmBOophJi9lElmJ1K2zKL_AxPoR6yvL9Y9vgzUrUGH4xEqetGRBQ0HS
Rabbit monoclonal anti-Jag1, CST, 2620S, human/mouse, WB/IF, RRID:AB_10693295, https://www.cellsignal.com/products/primary-antibodies/jagged1-28h8-rabbit-mab/2620?srsltid=AfmBOood4UntNGiRLGmTJkXmkhg9uCRus_STF1CSBwPROXfkK-iMn5Si
Rabbit monoclonal anti-LEF1, CST, 2230S, human/mouse/rat, WB/IF, RRID:AB_823558, https://www.cellsignal.com/products/primary-antibodies/lef1-c12a5-rabbit-mab/2230
Rabbit polyclonal anti-LHX1, Abcam, ab229474, mouse/rat, WB/IF/IHC, RRID:AB_2924798, https://www.abcam.com/en-us/products/primary-antibodies/lim1-lhx1-antibody-ab229474?srsltid=AfmBOornksY4oT8KPxYRT-VaqStClbcm0Mpos_4XMNphVLW4BZH9-xkx
Mouse monoclonal anti-MEIS1/2/3, Active Motif, #39795, human/mouse, IF/ICC, RRID:AB_2750570, https://www.activemotif.com/catalog/details/39795
Rabbit polyclonal anti-FN1, Proteintech, #15613-1-AP, human/mouse/rat, WB/IF/ICC/IP,RRID:AB_2105691, https://www.ptglab.com/products/FN1-Antibody-15613-1-AP.htm srsltid=AfmBOoo2NS59J5or4SVbrUwfhjbQwayzICx5nbCevZvl7LUjEMKNHjs
Goat polyclonal anti-ITGA8, R&D Systems, AF4076-SP, mouse/rat, WB/IF/ICC, https://www.rndsystems.com/products/mouse-rat-integrin-alpha8-antibody_af4076
Alexa Flour 488 goat anti-rabbit, Invitrogen, A11034, RRID:AB_2576217, https://www.thermofisher.com/antibody/product/Goat-anti-Rabbit-IgG-H-L-Highly-Cross-Adsorbed-Secondary-Antibody-Polyclonal/A-11034
Alexa Flour 488 goat anti-mouse, Invitrogen, A11001, RRID:AB_2534069, https://www.thermofisher.com/antibody/product/Goat-anti-Mouse-IgG-H-L-Cross-Adsorbed-Secondary-Antibody-Polyclonal/A-11001
Alexa Flour 488 donkey anti-goat, Invitrogen, A11055, RRID:AB_2534102, https://www.thermofisher.com/antibody/product/Donkey-anti-Goat-IgG-H-L-Cross-Adsorbed-Secondary-Antibody-Polyclonal/A-11055
Alexa Flour 546 goat anti-rabbit, Invitrogen, A11035, RRID:AB_2534093, https://www.thermofisher.com/antibody/product/Goat-anti-Rabbit-IgG-H-L-Highly-Cross-Adsorbed-Secondary-Antibody-Polyclonal/A-11035
Alexa Flour 546 goat anti-mouse, Invitrogen, A11030, RRID:AB_2737024, https://www.thermofisher.com/antibody/product/Goat-anti-Mouse-IgG-H-L-Highly-Cross-Adsorbed-Secondary-Antibody-Polyclonal/A-11030
Alexa Flour 546 goat anti-rat, Invitrogen, A11081, RRID:AB_2534125, https://www.thermofisher.com/antibody/product/Goat-anti-Rat-IgG-H-L-Cross-Adsorbed-Secondary-Antibody-Polyclonal/A-11081

Eukaryotic cell lines

Policy information about cell lines and Sex and Gender in Research

Cell line source(s)	HEK293T were purchased directly from ATCC (ATCC CRL-3216).
Authentication	Cell lines from ATCC were authenticated by STR profiling by the vendor.
Mycoplasma contamination	Cell live was mycoplasma free.
Commonly misidentified lines (See ICLAC register)	No misidentified cell lines were used.

Animals and other research organisms

Policy information about studies involving animals; ARRIVE guidelines recommended for reporting animal research, and Sex and Gender in Research

Laboratory animals	CMV-Cre stain (JAX stock # 006054), Six2-TGCtg BAC transgenic strain (JAX stock # 009606), Foxd1-GC strain (JAX stock # 012463) and the Rosa26 tdTomato reporter LSL-tdTtg strain Ai14D (JAX Stock # 007914) were purchased from the Jackson Laboratory. The conditional knock-in mouse models for Mllt1 T1-fl (ENL T1-fl) and Mllt1 T3-fl (ENL T3-fl) were generated in C57BL/6J background at the Jackson Laboratory using CRISPR/Cas9-mediated editing and homologous recombination. Timed mating was set up to collect kidneys at E14.5, E16.5, E18.5 and P0. Number of mice used in this study: 531.
Wild animals	No wild animals were used.
Reporting on sex	Both male and female mice were used in this study.
Field-collected samples	No field-collected samples were used.
Ethics oversight	All animal procedures were approved by the Institutional Animal Care and Use Committee (IACUC) at Van Andel Institute.

Note that full information on the approval of the study protocol must also be provided in the manuscript.

Plants

Seed stocks

Report on the source of all seed stocks or other plant material used. If applicable, state the seed stock centre and catalogue number. If plant specimens were collected from the field, describe the collection location, date and sampling procedures.

Novel plant genotypes

Describe the methods by which all novel plant genotypes were produced. This includes those generated by transgenic approaches, gene editing, chemical/radiation-based mutagenesis and hybridization. For transgenic lines, describe the transformation method, the number of independent lines analyzed and the generation upon which experiments were performed. For gene-edited lines, describe the editor used, the endogenous sequence targeted for editing, the targeting guide RNA sequence (if applicable) and how the editor was applied.

Authentication

Describe any authentication procedures for each seed stock used or novel genotype generated. Describe any experiments used to assess the effect of a mutation and, where applicable, how potential secondary effects (e.g. second site T-DNA insertions, mosiacism, off-target gene editing) were examined.

Flow Cytometry

Plots

Confirm that:

- [ ] The axis labels state the marker and fluorochrome used (e.g. CD4-FITC).
- [ ] The axis scales are clearly visible. Include numbers along axes only for bottom left plot of group (a 'group' is an analysis of identical markers).
- [ ] All plots are contour plots with outliers or pseudocolor plots.
- [ ] A numerical value for number of cells or percentage (with statistics) is provided.

Methodology

Sample preparation

Kidneys expressing tdTomato reporter were minced and treated with 0.25% trypsin or collagenase II to dissociate into single cell solution.

Instrument

BD FACSymphony S6 sorter was used to sort tdtomato+ cells.

Software

BD FACSDiva Software was used to sort tdtomato+ cells.

Cell population abundance

All tdTomato+ cells in embryonic kidneys were sorted. Most samples had 20-40% tdTomato+ cell population. The purity of sorted cells were assessed by BD FACSymphony S6.

Gating strategy

tdTomao+ cells were an well isolated population on the plot when sorting. Gating strategy is included in the Source Data file.

- [x] Tick this box to confirm that a figure exemplifying the gating strategy is provided in the Supplementary Information.
